# Inflammatory Aortopathies in Rheumatic Diseases: A State-of-the-Art Review

**DOI:** 10.3390/diagnostics16172709

**Published:** 2026-08-25

**Authors:** Mahmoud Abdelnabi, Nattanicha Chaisrimaneepan, Chanokporn Puchongmart, Ben Thiravetyan, Cristian Castillo-Rodriguez, Ramzi Ibrahim, Hoang Nhat Pham, Nouran Eshak, Megan M. Sullivan, Vivek Nagaraja, Brandon T. Larsen, Felipe Martinez, Ba D. Nguyen, Chadi Ayoub, Reza Arsanjani

**Affiliations:** 1 Department of Cardiovascular Medicine, Mayo Clinic, Phoenix, AZ 85054, USA; 2 Division of Rheumatology and Immunology, Department of Internal Medicine, University of Nebraska, Omaha, NE 68105, USA; 3 Department of Internal Medicine, Texas Tech University, Lubbock, TX 79409, USA; 4 Division of Cardiovascular Medicine, Department of Internal Medicine, University of Utah, Salt Lake City, UT 84132, USA; 5 Division of Rheumatology, Department of Internal Medicine, Mayo Clinic, Scottsdale, AZ 85259, USA; 6 Department of Laboratory Medicine and Pathology, Mayo Clinic, Phoenix, AZ 85054, USA; 7 Department of Radiology, Mayo Clinic, Phoenix, AZ 85054, USA

**Keywords:** aortopathies, aortitis, rheumatic diseases, aortic aneurysms, aortic dissection, aortic insufficiency, vascular complications, multidisciplinary care, rheumatoid arthritis, Takayasu arteritis, giant cell arteritis, systemic lupus erythematosus, imaging modalities, biologic therapy

## Abstract

Aortopathies in autoimmune rheumatic diseases (ARD) include a spectrum of aortic pathologies—including aortitis, aneurysms, dissections, and insufficiency—primarily caused by systemic inflammation. This comprehensive review investigates the clinical manifestations, pathophysiology, diagnostic modalities, and management strategies across various rheumatic diseases associated with aortopathies such as large vessel vasculitis (e.g., Takayasu arteritis, giant cell arteritis), connective tissue diseases (e.g., systemic lupus erythematosus, rheumatoid arthritis, ankylosing spondylitis, systemic sclerosis) and less common conditions (e.g., relapsing polychondritis, Cogan’s syndrome, Behçet’s disease, IgG4-related disease). Disease-specific pathophysiologic mechanisms of aortic wall inflammation and remodeling, including granulomatous and lymphoplasmacytic patterns and mixed inflammatory infiltrates, are described. Diagnostic imaging modalities—such as CTA, MRI, and PET/CT—are evaluated for their roles in detecting active inflammation, assessing structural complications, and guiding clinical decision-making. Histopathological findings provide insight into disease-specific vascular changes. Management strategies focus on the use of glucocorticoids, disease-modifying antirheumatic drugs (DMARDs), and biologics, including IL-6 and TNF-α inhibitors, with an emphasis on patient-centered approaches, multidisciplinary care, and timely surgical intervention for complications. Evidence gaps include optimal screening intervals and the role of novel biomarkers in risk stratification and in monitoring disease progression, highlighting the need for early recognition, frequent monitoring, and aggressive management of aortic involvement in rheumatic diseases to prevent life-threatening complications.

## 1. Introduction

Aortopathies in autoimmune rheumatic diseases (ARDs) refer to a spectrum of aortic involvement in various systemic inflammatory conditions. These conditions lead to inflammation of the aortic wall and its surrounding tissues, termed aortitis/periaortitis, at different degrees, rendering the aorta predisposed to dilation, aneurysm, dissection, or rupture [1,2]. Aortopathies are often well-recognized in large vessel vasculitides such as giant cell arteritis (GCA) or temporal arteritis and Takayasu arteritis (TA), according to the 2012 International Chapel Hill Consensus Conference (CHCC) [3]. Noninfectious inflammatory aortopathies have been occasionally reported in polymyalgia rheumatica (PMR), Behçet’s disease (BD), granulomatous with polyangiitis (GPA), Cogan syndrome, immunoglobulin G4-related disease (IgG4-RD), rheumatoid arthritis (RA), systemic lupus erythematosus (SLE), and sarcoidosis [1,2]. Other rheumatic diseases (RD) with aortic involvement are often under-recognized and not well addressed, but have a high mortality risk from complications such as aneurysmal rupture and dissection. Aortic disease may be a recognized manifestation of RD identified by screening using imaging (GCA, TA, occasionally PMR) or identified incidentally on imaging or detected on histology from aortic surgery or an autopsy [4,5,6]. Clinical presentation varies depending on the site of aortic involvement, such as back pain, abdominal pain, or acute aortic insufficiency if the aortic valve is involved. Patients can be asymptomatic or only have constitutional symptoms or present with life-threatening acute aortic syndromes such as aortic dissection, rupture, or intramural hematoma. Constitutional non-specific symptoms, including fever, malaise, and fatigue, are also possible [6,7]. The diagnosis requires a high index of suspicion of a certain rheumatic disease with a vague clinical presentation, especially in chronic or relapsing diseases [7]. Radiological, histopathological, and laboratory findings are necessary to establish the diagnosis, although, practically, tissue pathology is not always feasible in all cases [4,7].

Therefore, this review explores the understanding of aortopathies reported in rheumatic diseases.

**Scope and rationale.** For clarity, this review focuses on the inflammatory aortopathies that arise in rheumatic diseases, in which primary immune-mediated inflammation of the aortic wall or periaortic tissue (aortitis/periaortitis) predisposes to secondary structural complications such as dilatation, aneurysm, dissection and aortic regurgitation; degenerative and purely atherosclerotic aortopathies are outside its scope. Diseases were included when the published literature documented either (a) direct aortic wall or periaortic inflammation, (b) a reproducible association with clinically relevant secondary structural aortic complications, or (c) disease-specific diagnostic or therapeutic implications when aortic involvement occurs. The disorders are grouped as primary large-vessel vasculitides; other primary vasculitides; systemic autoimmune rheumatic diseases; autoinflammatory syndromes; and other immune-mediated fibro-inflammatory or granulomatous disorders. The review is evidence-based rather than exhaustive, and the strength of the underlying evidence varies substantially, ranging from cohort studies and guidelines to small case series and individual case reports.

## 2. Pathophysiology and Histopathology

**Figure 1:**
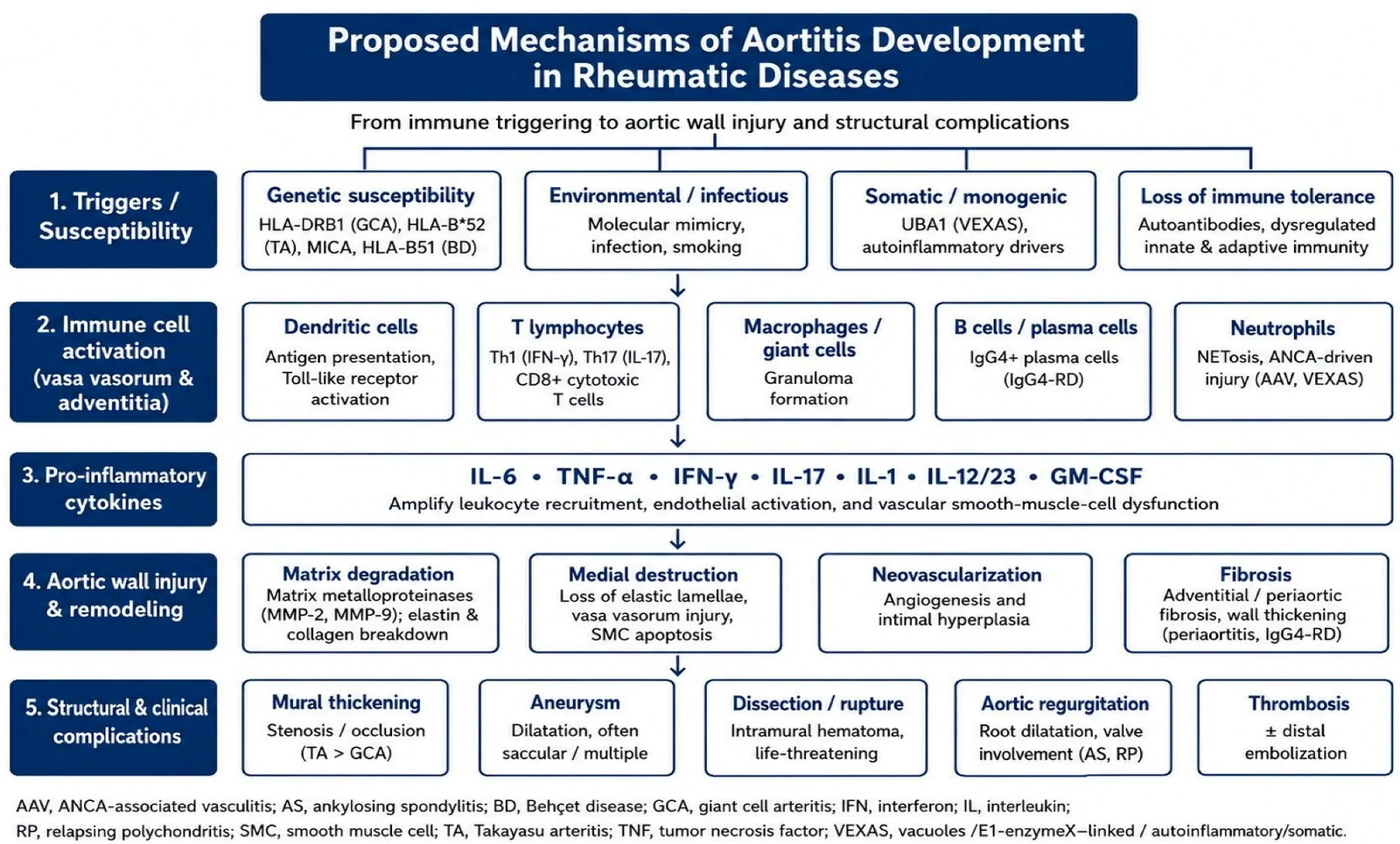
Proposed shared mechanisms in the development of aortitis in rheumatic diseases, from immune triggering and cell activation through cytokine-driven aortic wall injury to structural complications.

**Figure 2:**
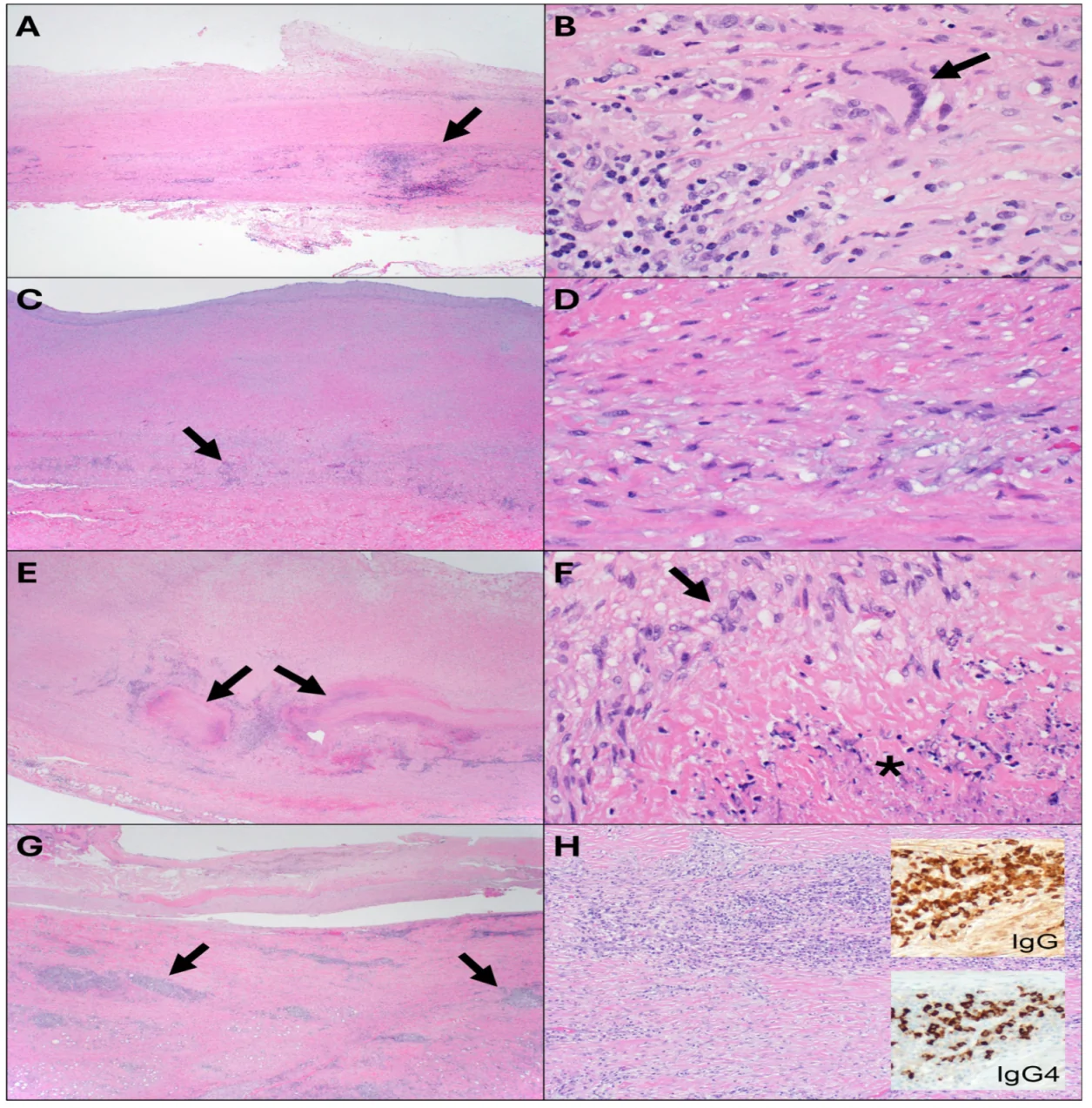
(**A**) In giant cell aortitis, lymphohistiocytic inflammation (arrow) destroys the media, and (**B**) scattered multinucleated giant cells are usually seen (arrow). (**C**) In late-stage Takayasu arteritis, the media and adventitia are densely fibrotic, with minimal residual chronic inflammation at the media–adventitia interface (arrow), and only a few lymphocytes are seen (**D**). (**E**) In granulomatosis with polyangiitis, granulomatous inflammation destroys the media (arrows) with (**F**) palisading histiocytes (arrow) surrounding geographic basophilic necrosis (*). (**G**) In IgG4-related disease, the adventitia and periadventitia show dense sclerotic fibroinflammatory change with lymphoid aggregates (arrows) that (**H**) are rich in plasma cells displaying an elevated IgG4: IgG ratio (>40%) by immunohistochemistry (insets). (**A**–**H**): Hematoxylin and eosin stains; original magnifications 20× (**A**,**C**,**E**,**G**), 100× (**H**), 200× ((**H**) insets), 400× (**B**,**D**,**F**).

The pathogenesis of aortic inflammation in elastic arteries varies across rheumatic conditions, resulting in distinct histopathological changes. Complications following damage to the elastic media include aneurysmal dilation, dissection, thrombosis with or without embolization, and aortic wall thickening leading to stenosis [8]. Inflammatory cell infiltration and subsequent destruction of the aortic tunica media and adventitia result in scarring and fibrosis over time, narrowing the lumen in some RDs, such as TA, to a greater extent than GCA. The destruction of the elastic lamina, inflammation in the vasa vasorum leading to vascular wall necrosis, and extensive inflammation in the media result in the formation of an aortic aneurysm [6]. The progression of an aortic aneurysm depends on the damage and interactions of all aortic constituents (smooth muscle cells, endothelial cells, macrophages, and fibroblasts) [9]. Aortitis secondary to ARDs often produces saccular/non-circumferential and multiple aneurysms [1]. Aortic dissection occurs due to intimal tears that allow blood into the media, separating the elastic lamellae. Rupture occurs when the dissection extends through the adventitia and can be aneurysmal or non-aneurysmal [1,8]. Aortitis/periaortitis is classified based on the pattern of inflammation, which is not attributable to atherosclerosis. In aortitis, inflammation affects the media and/or intima, often extending to the adventitia, and can impact any segment of the aorta, including its major branches or other large arteries [4]. Periaortitis refers to an inflammatory condition originating from the adventitia of the aortic wall, extending into the surrounding periaortic space as seen in IgG4-RD [10]. Although the definitions of both entities are distinct, there can be significant overlaps in their presentation, particularly when aortitis extends into the PA or vice versa. Table 1a categorizes Rheumatic diseases according to the pattern of inflammation. Table 1b summarizes the histopathological findings of aortitis/periarteritis related to rheumatic diseases. Figure 1 illustrates proposed mechanisms of aortitis development and Figure 2 illustrates the histopathology of aortitis in the setting of representative rheumatic diseases.

## 3. Aortic Involvement in Rheumatic Diseases

### 3.1. Takayasu Arteritis (TA)

Takayasu arteritis (TA) is a chronic inflammatory condition affecting large blood vessels, causing stenosis or occlusion in 98% and aneurysms in 27% of individuals’ blood vessels [11], particularly involving the aorta and its major branches [12]. TA is characterized by pathological changes in large blood vessel layers, including arterial wall thickening, stenosis, occlusion, aneurysm formation, and dissection [12]. In some, it may initially present as aortic dissection after childbirth in women in their 30s or as a myocardial infarction [11,13,14]. TA is currently classified into six types, as shown in Table 2.

**Figure 3:**
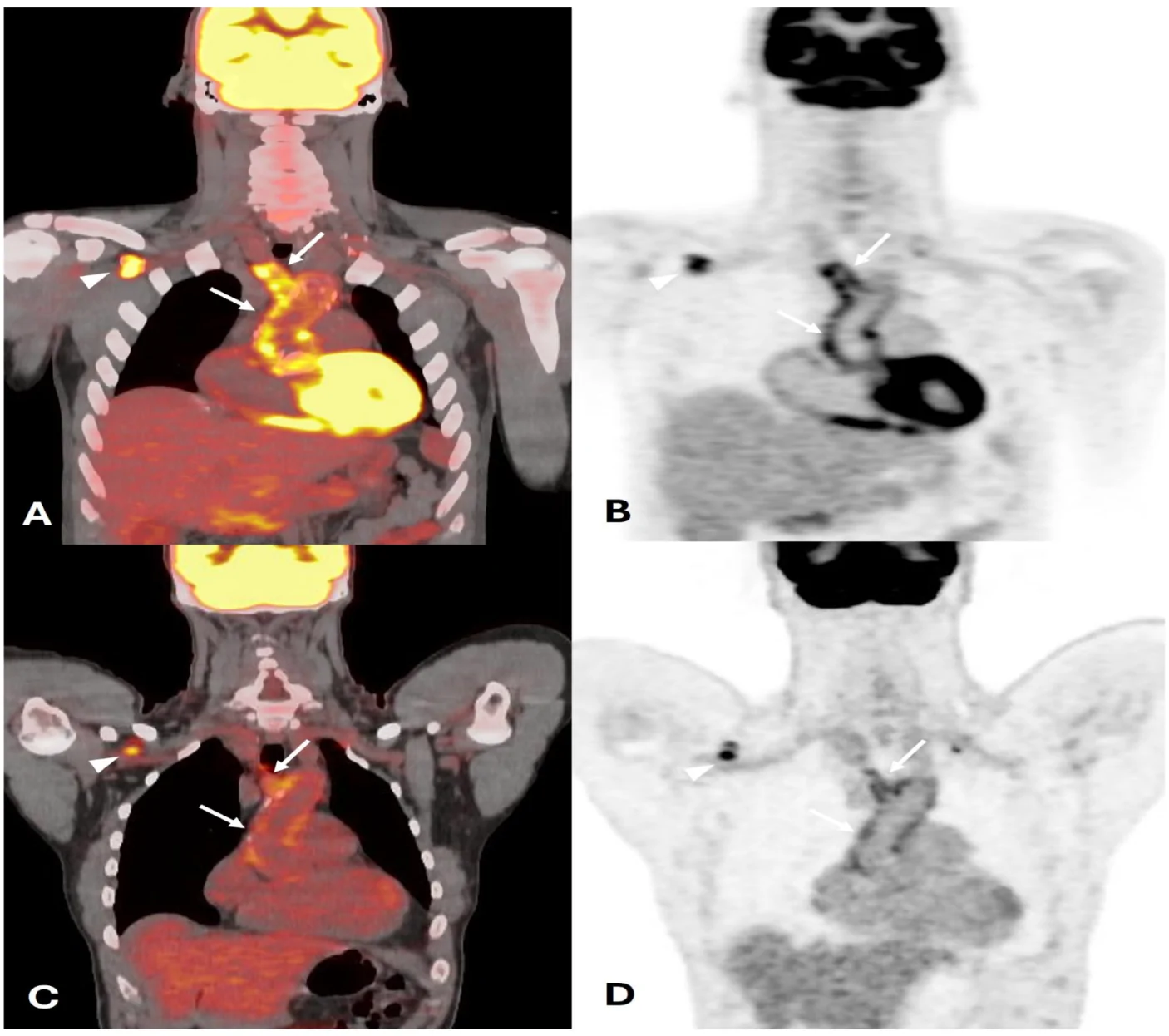
An example of Takayasu’s disease status post ascending aorta tubular grafting with recurrent vasculitis. Coronal fused and attenuation corrected PET images of F-18 FDG PET/CT studies at 2 different time points before and after immunosuppressive therapy with high-dose glucocorticoids. Pretherapy images (**A**,**B**) show intense abnormal radiotracer uptake throughout the aortic root, ascending aorta graft, brachiocephalic artery, and aortic arch (arrows) consistent with active vasculitis. Post-therapy images (**C**,**D**) show significantly improved radiotracer uptake involving the same vessels, consistent with decreased inflammation (arrows). In addition, focal tracer activity in the distal right subclavian artery (arrowheads) also improved after initiation of therapy.

**Figure 4:**
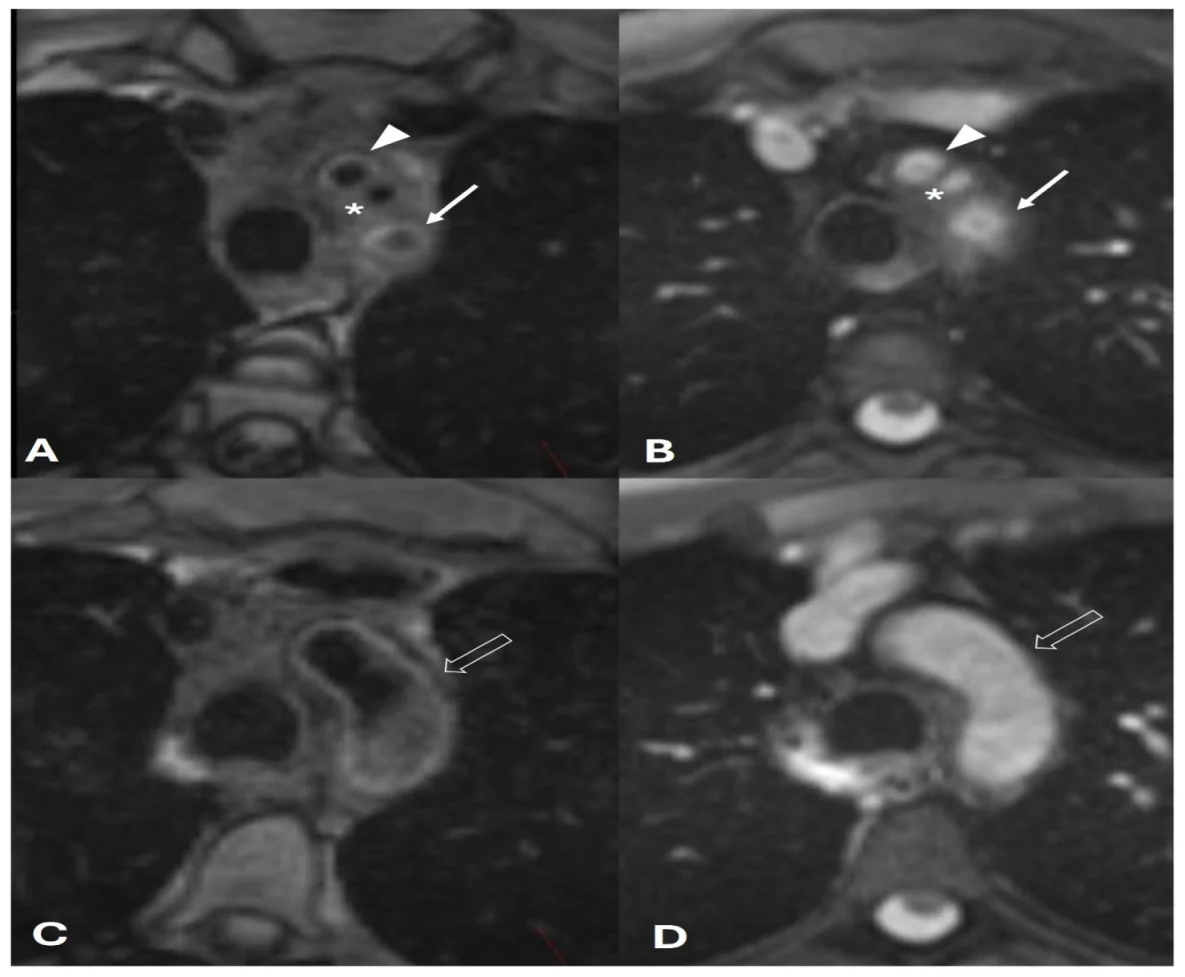
An example of known Takayasu’s disease evaluated for abnormal and asymmetric peripheral pulses. Axial STIR images of chest MR without contrast (**A**,**C**) and axial images of chest MRA with contrast (**B**,**D**) showing near concentric mural thickening and edema in the proximal left subclavian artery resulting in luminal narrowing (arrows) and to a lesser extent thickening and edema in the aortic arch (open arrow), right brachiocephalic artery (arrowhead) and left common carotid artery (*).

**Figure 5:**
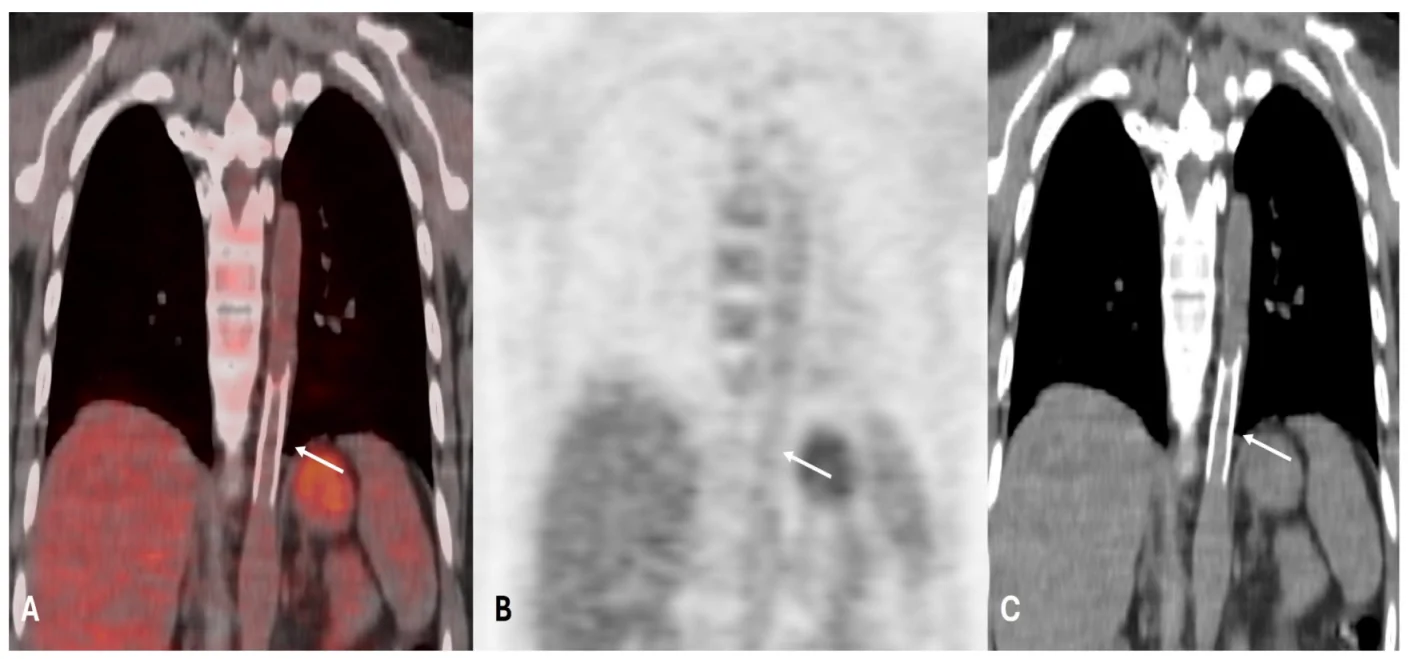
An example of known Takayasu’s disease status post descending aorta stent placement on remission. Coronal images of F-18 FDG PET/CT (**A**) fused, (**B**) AC PET and (**C**) CT without contrast showing a distal descending aorta stent with residual mild luminal narrowing (arrows). There is normal radiotracer distribution with no pathological aortic wall activity to suggest active inflammation.

**Figure 6:**
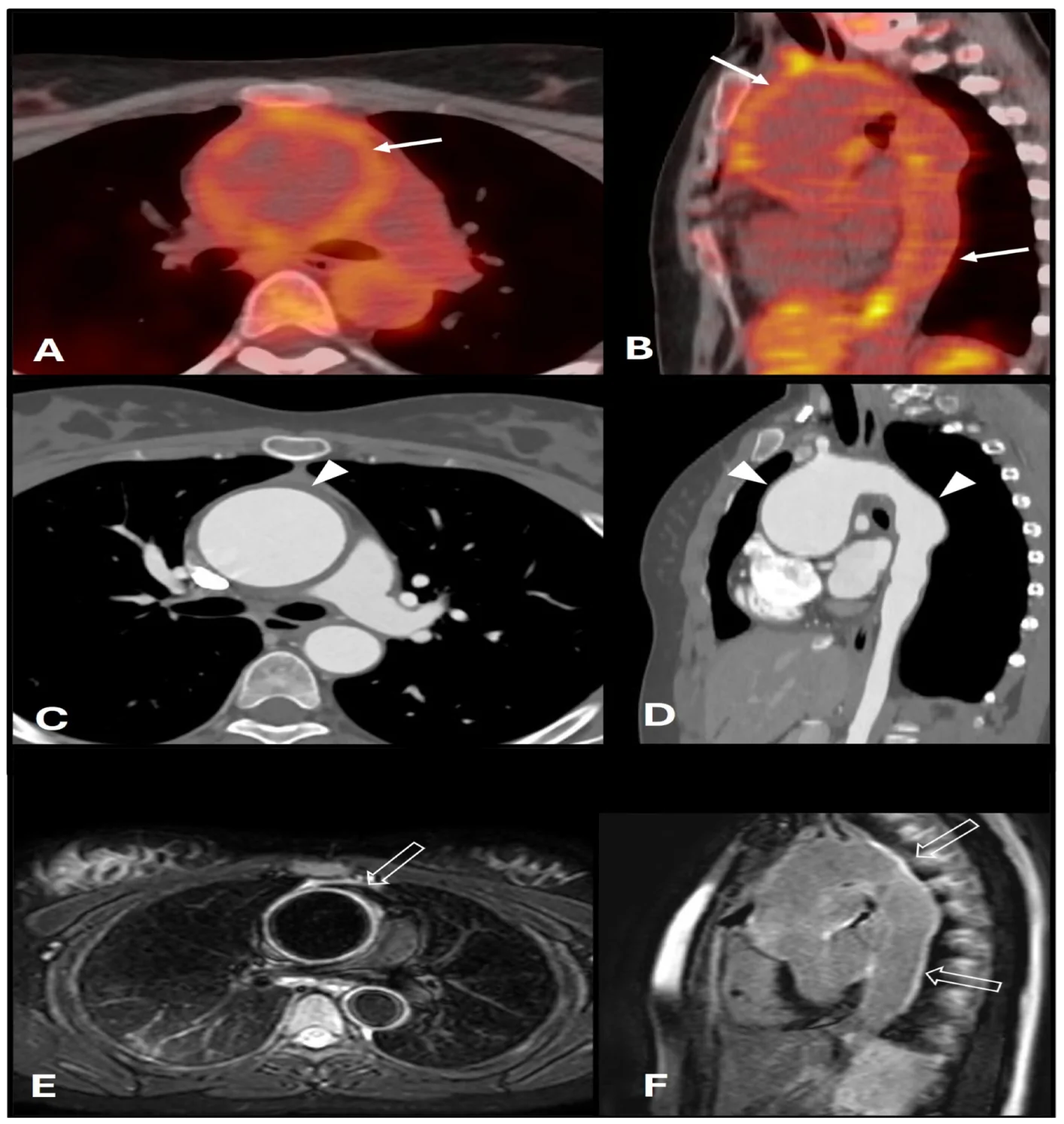
Takayasu’s disease with aortic involvement and thoracic aorta aneurysm off therapy evaluated for disease activity. Axial (**A**) and sagittal (**B**) fused images of F-18 FDG PET/CT showing extensive pathological tracer activity throughout a dilated thoracic aorta (arrows) involving predominantly the ascending aorta to a lesser extent arch and descending aorta most consistent with active vasculitis. Axial (**C**) and sagittal (**D**) images of CTA of the chest with IV contrast showing aneurysmal dilation to the ascending and to a lesser extent descending aorta associated to diffuse mural thickening (arrowheads). Axial STIR (**E**) images of chest MR showing diffuse aortic wall edema (open arrow). Sagittal double inversion recovery images after IV gadolinium administration (**F**) of chest MR showing diffuse thoracic aorta wall hyperenhancement (open arrow) consistent with active inflammation.

These classifications are essential for understanding clinical variations and informing the management strategies of affected patients. The pathogenesis of TA is not well understood [15]. The T-cell-mediated pathway is believed to play a major role in the development of TA. Specific T cells, such as vasculogenic T cells, particularly Th1 and Th17, are dominant in the vascular layers, causing vasculitis and organ damage [16]. Their activity is closely associated with the presence of interleukin-6 (IL-6) and interleukin-17 (IL-17) [17]. Activation of natural killer cells, γ/δ T cells, and macrophages with the production of tumor necrosis factor-α (TNF-α), forming granulomas within the blood vessels [18]. The diagnosis of aortic involvement in TA is essentially made with imaging techniques guided by a high degree of clinical suspicion. Imaging studies also help in determining the extent of vascular involvement. There are two key aspects to imaging of the arterial tree in TA and other large-vessel vasculitides: evaluation of the arterial lumen and evaluation of the arterial wall, with the acknowledgment that arterial wall biopsy is seldom performed. To this effect, magnetic resonance angiography (MRA) or computed tomography angiography (CTA) demonstrates smoothly tapered luminal narrowing or occlusion of the affected vessel. Three-dimensional reconstruction protocols are available to examine the vessel wall in greater detail. Positron emission tomography (PET) in combination with computed tomography (CT) or magnetic resonance (MR) is increasingly used to identify areas of increased uptake, or “hot segments,” in the vessel wall (reflecting higher standardized uptake values) to indicate active vasculitis in the right clinical setting [17,19]. The initial change observed is mural thickening, indicating inflammation of the media and adventitia, followed by structural vascular changes detected on CTA [20]. Aneurysms are less common and typically affect the ascending aorta and abdominal aorta [21]. However, among these modalities, CTA is the most favored diagnostic tool due to its ability to assess disease severity, its widespread availability, and its high-quality images for follow-up of the affected vessels, stents, and bypass grafts [17,22]. The goal of treatment is to eliminate or reduce inflammatory activity in the vessel wall using immunosuppressive therapy (IST) and to limit further structural abnormalities in the vessels. Glucocorticoids (GCs) are effective for most patients; however, given the chronic, relapsing nature of the disease in more than half of TA patients, the use of GC-sparing therapy (also called IST in this setting) is imperative at an early stage [11]. In most cases, upfront use of GC with IST provides better control of disease activity than GC alone [11]. Initial options for IST include methotrexate (MTX) and azathioprine (AZA), with alternatives such as mycophenolate mofetil (MMF) or leflunomide. Unfortunately, relapse is common when tapering prednisone to dosages of 15 mg/day or less [23]. In selected or treatment-refractory cases, tumor necrosis factor alpha inhibitors (TNFi) are used. More severe diseases may respond to TNF inhibition or IL-6 inhibition [24]. Those with severe vascular complications may require surgical evaluation and intervention. Revascularization for TA has been shown to be safe and is associated with low morbidity and mortality rates [23]. Conventional bypass grafts typically achieve optimal long-term outcomes. Additionally, percutaneous transluminal angioplasty has yielded favorable results, particularly for short lesions [23] (Figure 3, Figure 4, Figure 5 and Figure 6).

### 3.2. Giant Cell Arteritis (GCA) and Polymyalgia Rheumatica (PMR)

**Figure 7:**
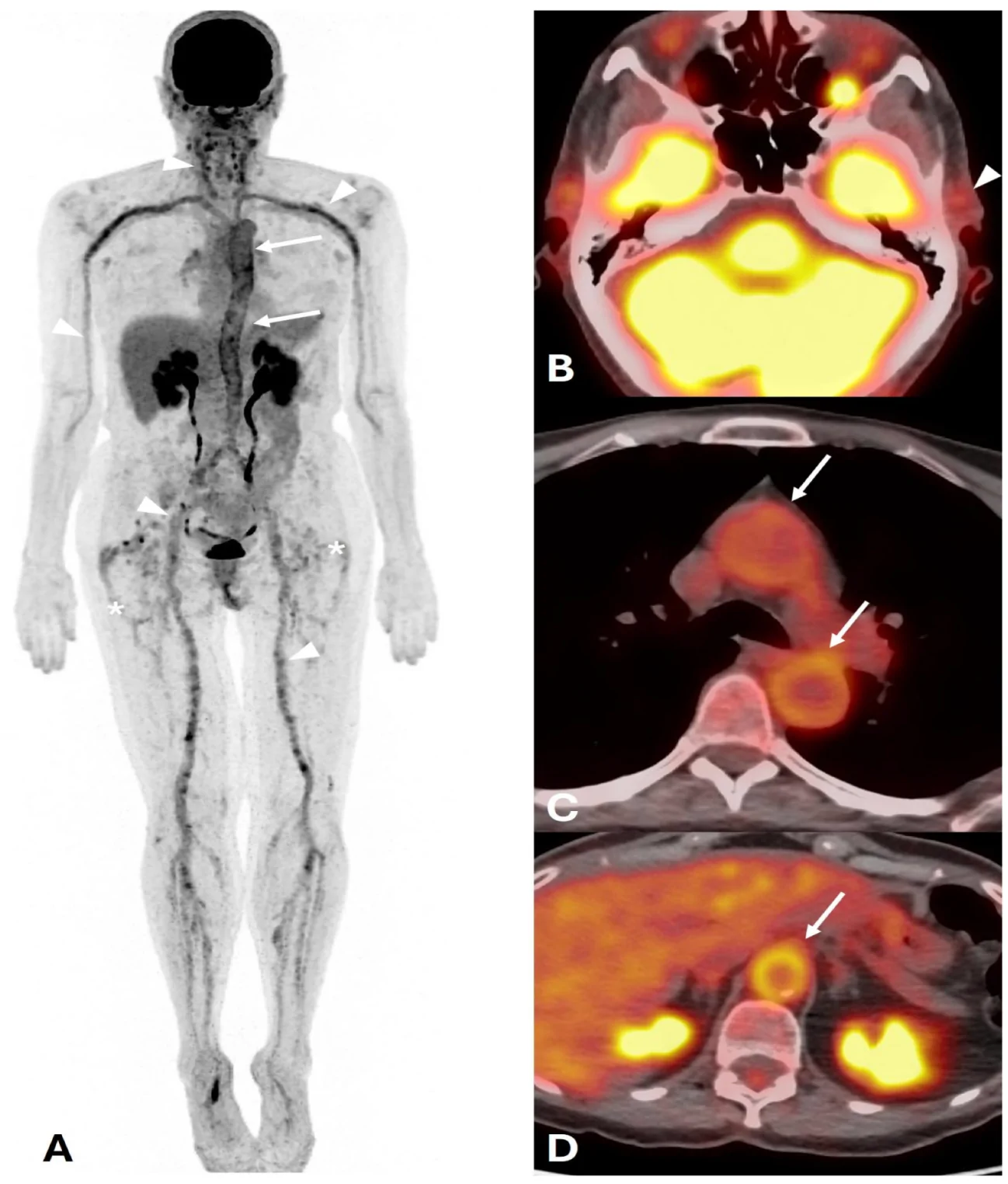
Patient with known giant cell arteritis. Images of whole-body F-18 FDG PET/CT, PET MIP (**A**) and axial fused images at multiple levels (**B**–**D**) showing extensive abnormal tracer activity involving the aorta (arrows), as well as head and neck vessels, upper and lower extremity arteries and superficial temporal arteries (arrowheads) indicating active vasculitis. In addition, multifocal areas of abnormal tracer accumulation in lower extremities musculature most consistent with active myositis (*).

**Figure 8:**
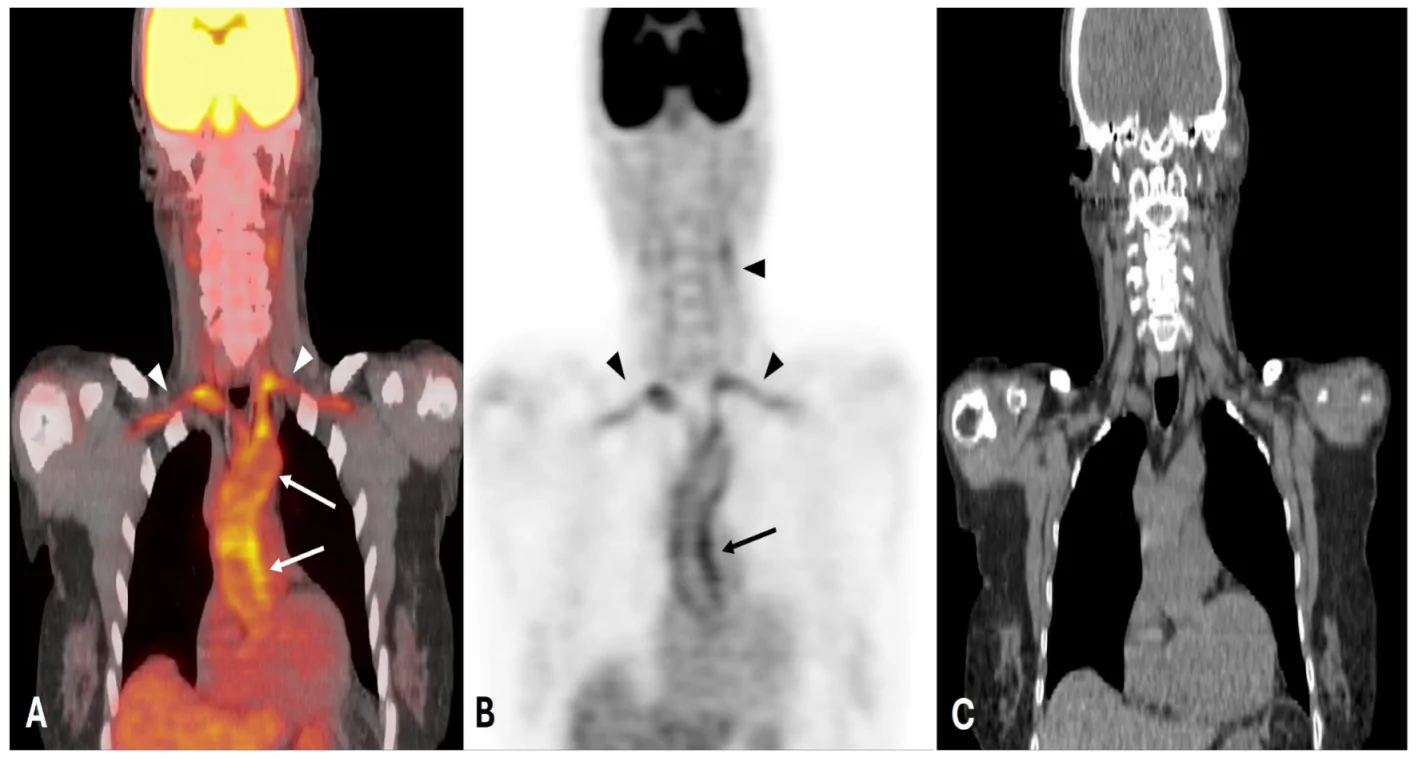
An example of a known giant cell arteritis. Coronal images of F-18 FDG PET/CT scan (**A**) fused, (**B**) AC PET,(**C**) CT without contrast showing intense tracer accumulation throughout the ascending aorta and aortic arch (arrows) as well as right brachiocephalic artery, bilateral subclavian arteries and carotid arteries (arrowheads) indicating active vasculitis.

**Figure 9:**
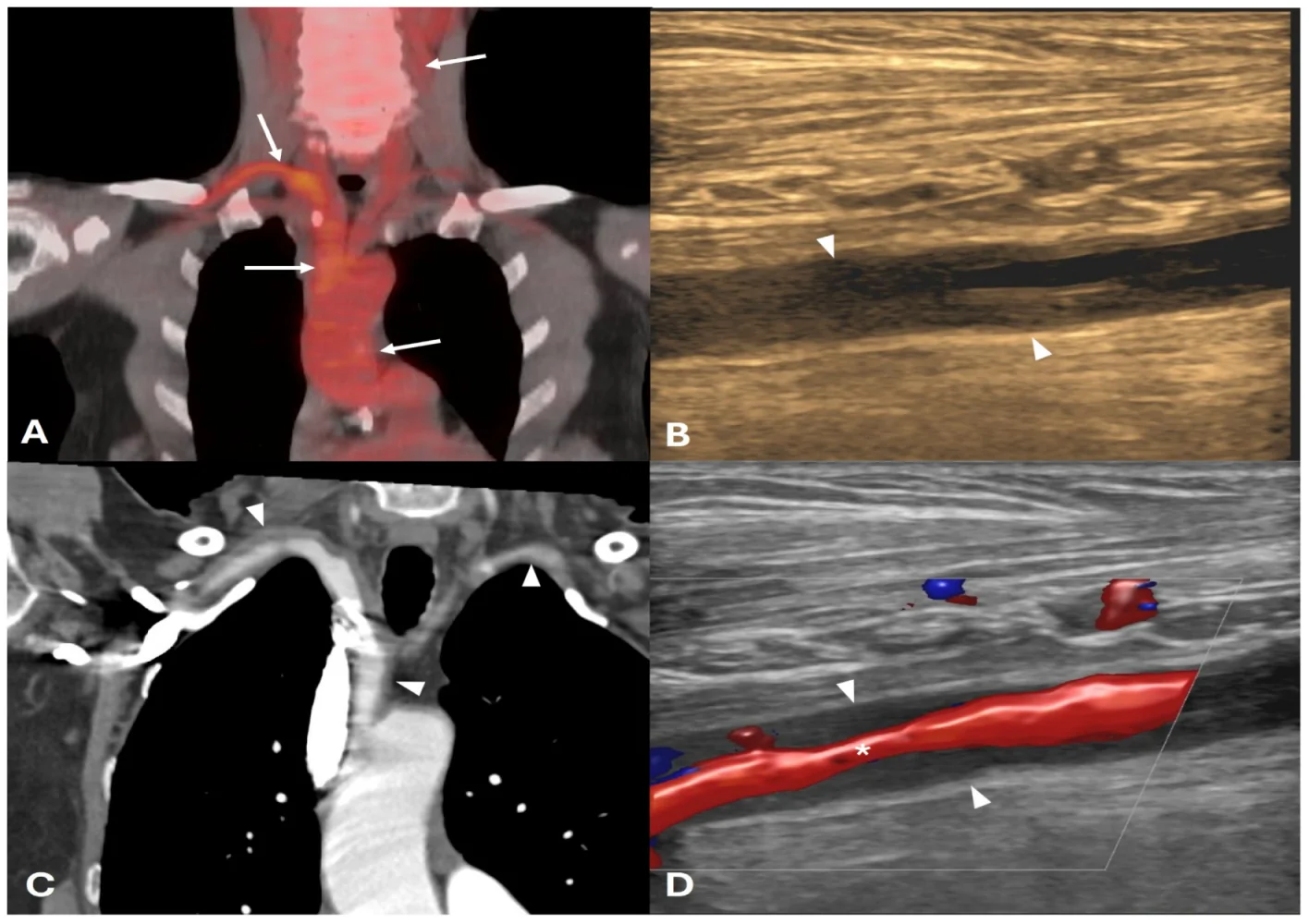
An example of known giant cell arteritis on therapy with elevated inflammatory markers evaluated for disease activity. Coronal fused MPR image of F-18 FDG PET/CT (**A**,**C**), gray scale (**B**) and color Doppler (**D**) ultrasound images of the right subclavian artery and coronal MPR images of chest CTA. There is abnormal radiotracer uptake involving the ascending aorta, right brachiocephalic, bilateral subclavian and to a lesser extent bilateral common carotid arteries (arrows). In addition, evidence of concentric mural thickening to the brachiocephalic and bilateral subclavian arteries (arrowheads). In the right subclavian artery, there is moderate luminal narrowing (arrowheads), turbulent flow on color doppler (*).

**Figure 10:**
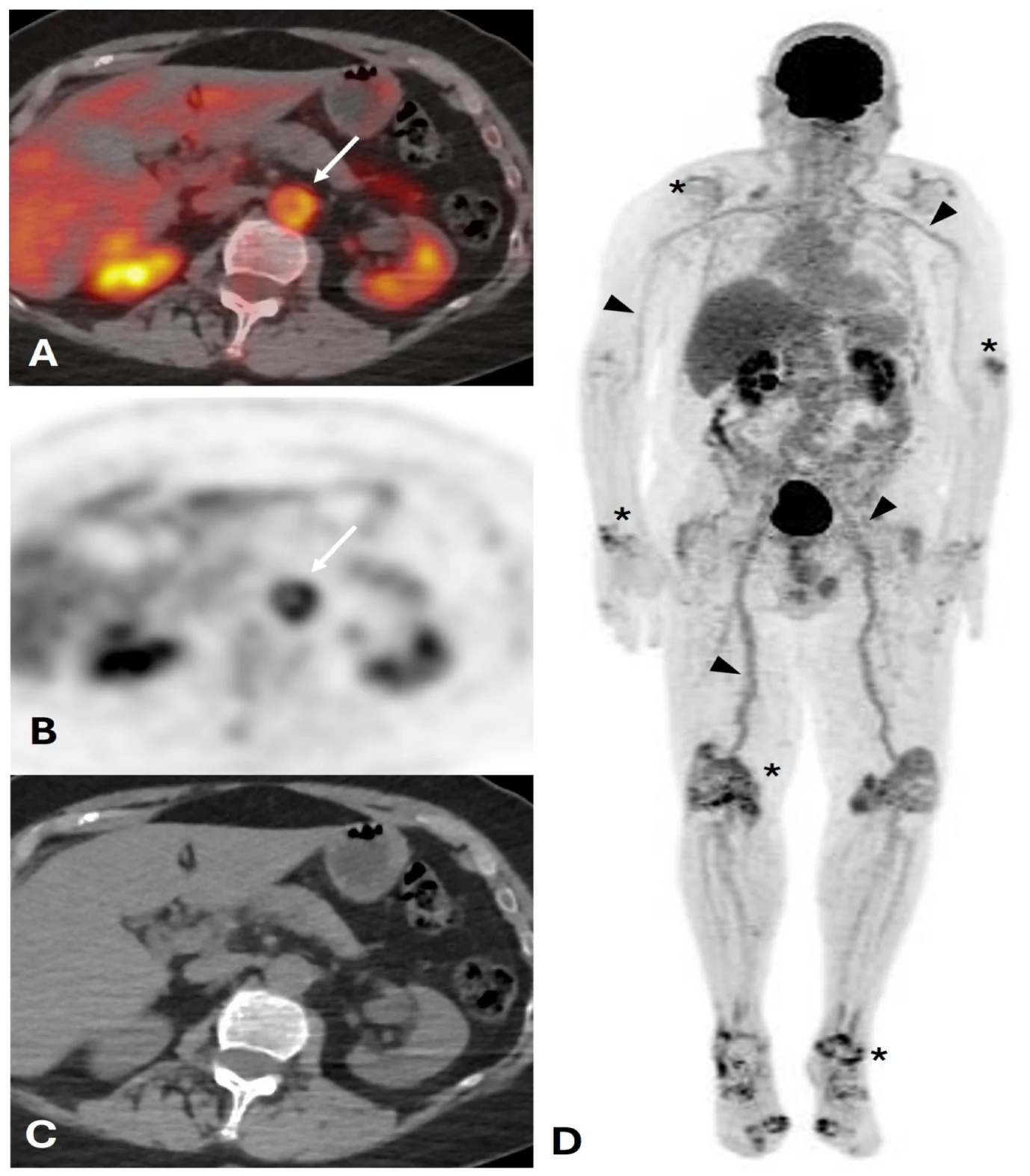
Whole body F-18 FDG PET/CT in a patient with known polymyalgia rheumatica evaluated for persistently elevated inflammatory markers. Axial fused (**A**), axial AC PET (**B**), axial CT without contrast (**C**) and whole-body AC PET MIP (**D**) images showing abnormal tracer uptake throughout the abdominal aorta (arrows) and to a lesser extent bilateral subclavian, axillary, brachial, iliac and femoral arteries (arrowheads) consistent with active vasculitis. In addition, heterogeneous moderate to intense abnormal tracer accumulation involving bilateral shoulders, elbows, wrists, hands, knees, ankles and feet (*) most consistent with active inflammatory polyarthritis.

Giant cell arteritis (GCA) is the most common form of systemic large vessel vasculitis (LVV). GCA and PMR are closely related conditions, often considered different clinical manifestations of the same diagnostic and pathogenic spectrum. During their course, 40–60% of patients with GCA will have PMR, whereas 16–21% of patients with PMR will have GCA [25]. PMR large-vessel involvement, consistent with “subclinical GCA,” is present in 30%. The aortitis itself is not attributed to PMR and is typically considered a missed diagnosis of GCA [26]. Figure 7, Figure 8 and Figure 9 demonstrate imaging findings of the aorta in GCA, and Figure 9 demonstrates imaging findings in a case of PMR. Unlike PMR, GCA is the leading cause of aortitis, accounting for 72% of patients [2,3,4,5]. Aortitis can occur at any stage of the disease onset [2,3,4,5], and often presents silently or with minimal constitutional symptoms at the time of diagnosis [18,27]. Its complications include aortic insufficiency, ruptured aortic aneurysm, aortic dissection, stroke, or myocardial infarction [28]. Among these, the most common aortic complication was found to be an aortic aneurysm (up to 20%) [29]. The prevalence of thoracic aortic aneurysms ranges from 2–18% among patients with GCA [30,31]. A previous retrospective study involving 96 GCA patients showed a 17.3-fold increase in the risk of developing a thoracic aortic aneurysm and a 2.4-fold increase in the risk of an isolated abdominal aneurysm [32]. Predictors of mortality among GCA patients with aortitis include the presence of aortitis or any aortic involvement at disease onset, as well as the presence of symptoms [33,34,35]. Diagnostic imaging modalities for detecting GCA aortic involvement include arteriography [32], CTA, MRA, and FDG-PET [36]. Aortitis was detected in up to 65% of cases on CTA screening, and follow-up aortic screening is recommended to detect stenosis and aneurysms [37]. The reported sensitivity and specificity of PET in the diagnosis of GCA are 88.9% and 95.1%, respectively [32], though these values depend on the timing of glucocorticoid initiation [38]. Generally, a PET scan detects the uptake up to 51–65% at the thoracic aorta and 54% at the abdominal aorta in GCA patients, reflecting ongoing inflammation in those with subclinical aortic inflammation [39,40]. However, large-vessel involvement often goes undetected at early disease onset, whether on MRA or PET scans [18]. Due to the increasing body of evidence on the high prevalence of large vessel vasculitis in GCA, the 2021 American College of Rheumatology (ACR)/Vasculitis Foundation guidelines for management of GCA conditionally recommend obtaining noninvasive vascular imaging (MRA or CTA of the neck/chest/abdomen/pelvis) to evaluate large vessel involvement at the time of diagnosis [24]. While there is no consensus on monitoring guidelines for patients with large-vessel vasculitis, proactive and periodic monitoring using vascular imaging may be needed to identify complications [36]. Given the considerable morbidity and mortality associated with aortic aneurysms, the aorta should be evaluated routinely during the follow-up of patients with GCA. The initial treatment for GCA-associated inflammatory aortitis is high-dose GC, dosing 40–60 mg once daily or 0.7 mg/kg/day with gradual tapering [6,41]. Concurrent initiation of GC-sparing therapy is advised, given the anticipated prolonged duration of GC therapy, which predisposes to side effects, and the accumulating body of evidence with interleukin-6 targeted therapy (tocilizumab). Tocilizumab was recently used as a treatment and is now considered a first-line therapy, in combination with glucocorticoids, per the 2021 ACR management guidelines [24,42,43,44]. While tocilizumab is favored, methotrexate can be considered as an alternative option for patients unable to use tocilizumab due to factors such as recurrent infections, history of gastrointestinal perforations or diverticulitis, and cost. Aortic aneurysms may initiate the disease several years after the diagnosis, occasionally after corticosteroid therapy discontinuation. Aortic ectasia and aneurysms should be closely monitored to determine the appropriate timing for surgical intervention [41]. There is no consensus on the optimal imaging modalities or the frequency of sequential imaging. Imaging screening is recommended at regular intervals over the long term because, despite treatment, 56% still had evidence of active vasculitis of the thoracic vessels by PET and 80% by MRI [32,45]. Despite apparent clinical and biomarker remission, up to 63–84% of scans show persistent uptake on PET [30]. However, after 1 year of corticosteroid treatment, contrast enhancement resolved in 93.75% of cases in GCA patients [30]. However, a minor relapse was found during the 2-year follow-up [44] (Figure 7, Figure 8, Figure 9 and Figure 10).

### 3.3. Kawasaki Disease

Kawasaki disease is a vasculitis of unknown cause that primarily affects children under the age of 5 years old. It commonly involves inflammation of medium-sized arteries; thus, aortic involvement is rare. A prospective cohort study of 115 patients with Kawasaki disease in Brazil found that 1% had thoracic aorta aneurysms (TAA) [46]. The complications were more common in patients with persistently elevated inflammatory markers and those who received IVIg after the first 10 days of disease onset [46]. Another cohort from Singapore found that despite early treatment with corticosteroids and IVIg, patients who had prolonged CRP elevation for more than 2 weeks, relapsing symptoms, and prolonged admission were associated with aortitis [47]. MRA, in addition to transthoracic echocardiogram (TTE), could be considered for evaluating large-vessel arteritis in these patients [47]. Treatment of Kawasaki disease typically involves a combination of aspirin and high-dose IVIg as soon as possible after diagnosis [48]. Untreated childhood Kawasaki disease could present with an ascending aortic aneurysm in addition to a coronary artery aneurysm later in life [49]. Prompt recognition and early treatment with IVIg could potentially reduce the risk of cardiac and aortic involvement in patients with Kawasaki disease.

### 3.4. Antineutrophilic-Cytoplasmic Antibodies (ANCA) Associated Vasculitis (AAV)

AAV with aortic involvement is uncommon, and the incidence is unknown. Less than 20 cases of aortic involvement in AAV, which includes granulomatosis with polyangiitis (GPA), microscopic polyangiitis (MPA), and eosinophilic granulomatosis with polyangiitis (EGPA), have been reported [50,51]. ANCA-related aortic involvement can present with aortic aneurysm, aortic dissection or rupture, and aortic insufficiency as a consequence of aortitis [51,52] and can precede the clinical manifestation of AAV [53,54]. Both the thoracic and abdominal aorta may be involved; some present with PA detected on imaging [4]. An observational study by Kaymakci et al. demonstrated that most patients (*n* = 25, 69%) were diagnosed with large-vessel vasculitis and AAV within a 1-year period. Most (69%) had histopathological confirmation of an AAV diagnosis in a location other than the temporal artery, the aorta, or the periaortic soft tissue. Seventeen patients had TA-AAV, 10 had aorta-AAV, and 9 had PA-AAV [55]. Clinical symptoms, imaging, and immunologic tests may be required for the diagnosis [56]. Documented possible pathogeneses of aortitis in AAV are vasculitis of vasa vasorum in the adventitial layer, progression from initial intimal injury to transmural aortitis, and peri-aortitis, which eventually causes aortic inflammation [51,53,57,58,59]. Some patients remain asymptomatic, but aortitis was detected on FDG-PET/CT [60]. Treatment with corticosteroids and cyclophosphamide or rituximab has been reportedly successful for remission induction. Surgery is indicated for aortic aneurysms or dissections [61].

### 3.5. Immunoglobulin G4-Related Diseases (IgG4-RD)

**Figure 11:**
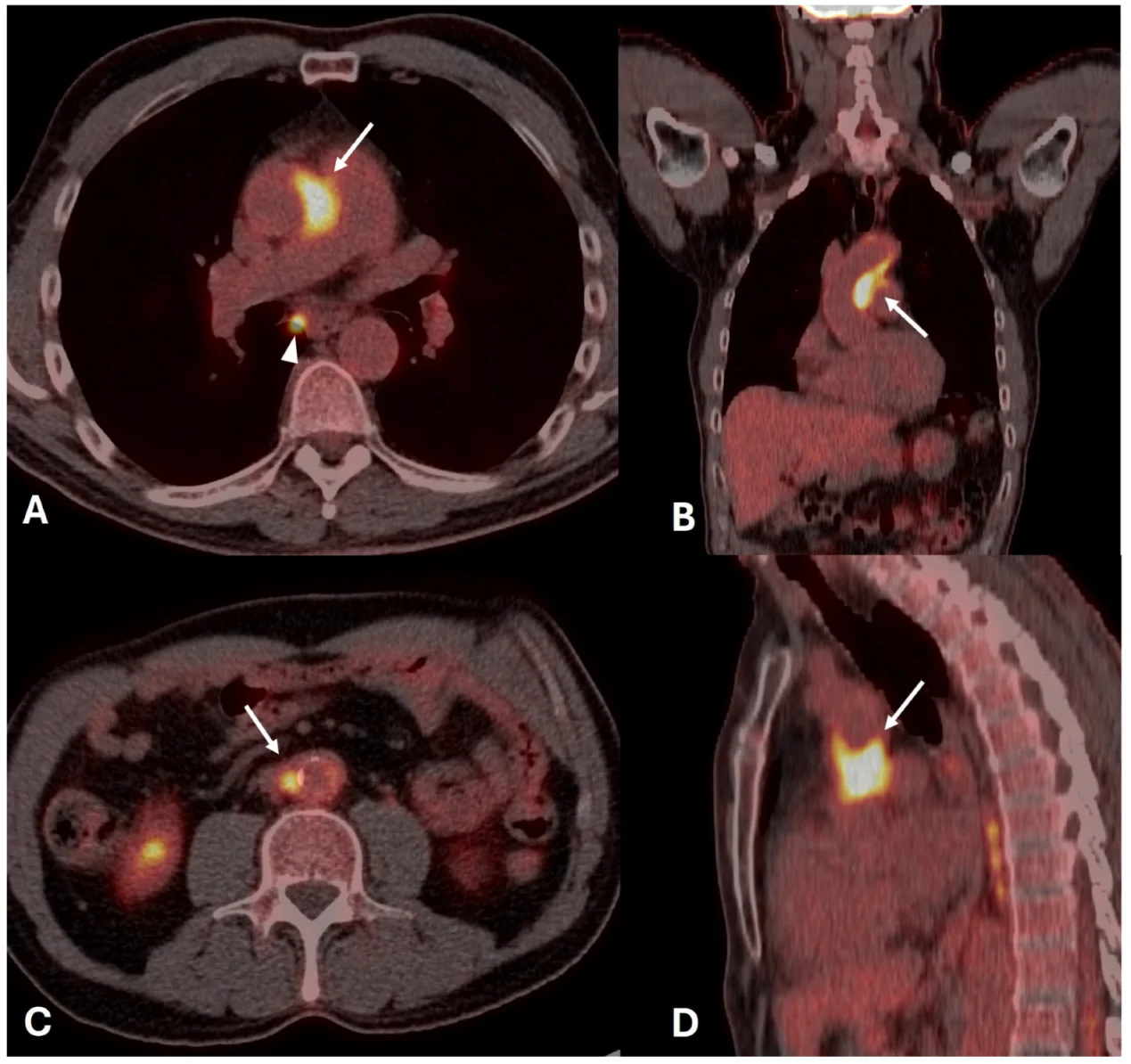
An example of known IgG4 disease evaluated for persistent elevated inflammatory markers. Axial (**A**,**C**), coronal (**B**) and sagittal (**D**) images of F-18 FDG PET/CT showing tracer avid mural thickening involving the ascending aorta, proximal aortic arch, and upper abdominal aorta (arrows) indicating active vasculitis. There is a subcentimeter tracer avid mediastinal level 7 lymph node (arrowhead) which is likely inflammatory.

**Figure 12:**
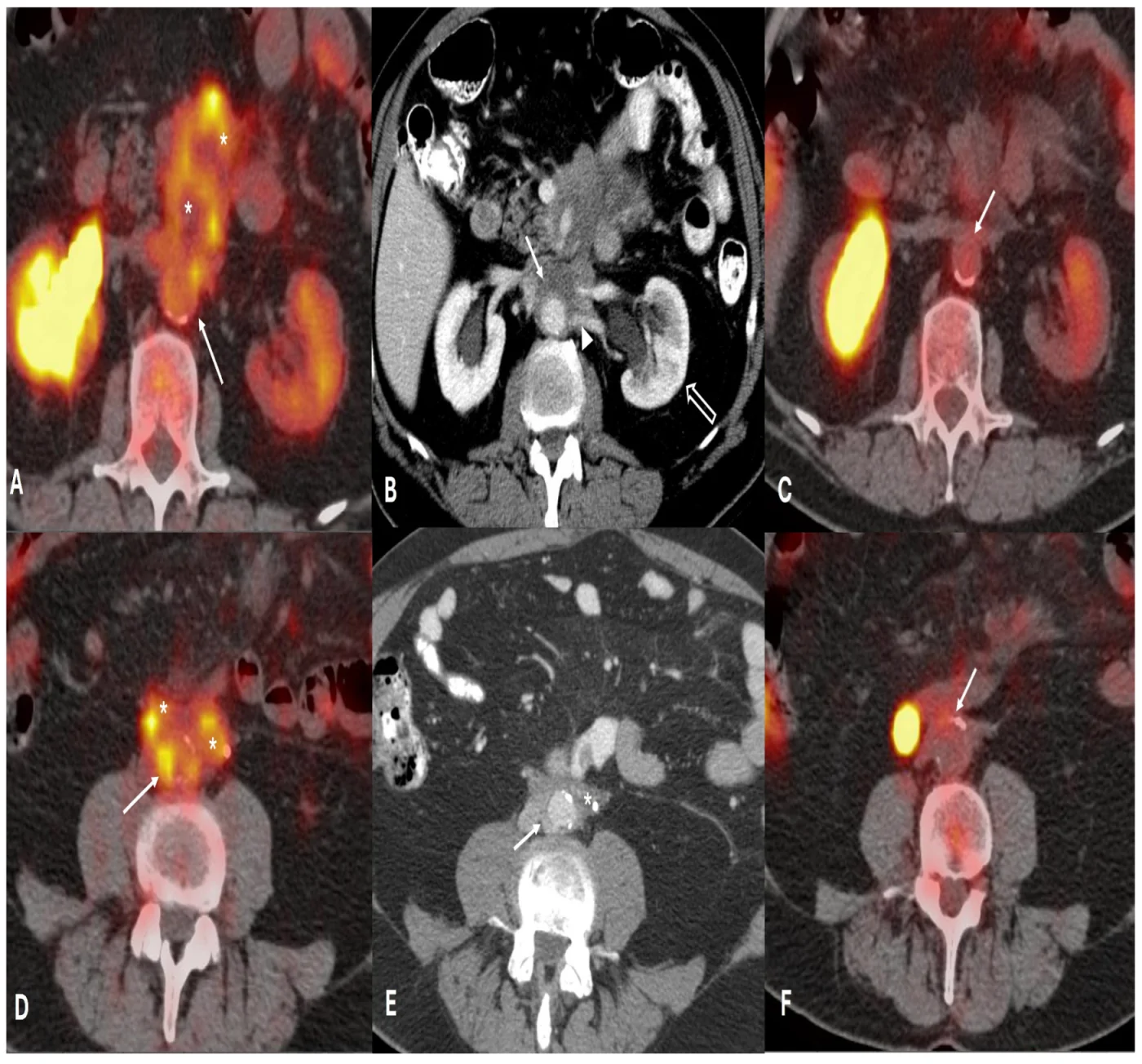
An example of known IgG4 disease with active vasculitis. Imaging before and after immunosuppressive therapy. Pretherapy images of fused axial F-18 FDG PET/CT at the level of the kidneys (**A**) and just above the bifurcation (**D**) and CT scan of the abdomen with oral and IV contrast at the same levels (**B**,**E**) showing tracer avid mural thickening to the abdominal aorta (arrows) and ostial portion of the left renal artery (arrowhead) associated to tracer avid mesenteric and retroperitoneal perivascular soft tissue mass (*) consistent with active vasculitis and surrounding inflammation. Please also note decreased perfusion to the left kidney (open arrow) secondary to ostial narrowing. Post therapy images of fused axial F-18 FDG PET/CT (**C**,**F**) showing near complete resolution of previously noted tracer avid vasculitis with surrounding inflammatory changes (arrows).

IgG4-RD is a systemic fibro-inflammatory condition involving several organs. Cardiovascular involvement frequently presents as aortitis, predominantly involving the abdominal aorta [62]. Patients are usually asymptomatic; the condition is often detected incidentally on imaging or histopathologically after aortic surgery [63]. Clinical symptoms depend on the site of aortic involvement and may include back pain, abdominal pain, and lower-extremity edema; constitutional symptoms such as fever or night sweats are rare, as is a significant increase in CRP [64,65]. Chronic peri-aortitis (CP) is more common and is associated with peripheral tissue involvement and retroperitoneal fibrosis (RPF); features overlapping with aortitis can also occur [64,66]. Aortic involvement is found in approximately 10% of IgG4-RD and is more common in men [67]. Aortitis can be complicated by aneurysm formation and dilatation and, less frequently, dissection [68,69,70]. Table 3 summarizes the features of IgG4-aortitis and IgG4-CP [71]. The pathogenesis of aortic disease is unknown, but it is presumed to be mediated by B cells and cytotoxic CD4 T cells [67,72]. Thoracic aortitis is twice as common as abdominal aortitis but with less vascular wall thickening, while chronic peri-aortitis affects the abdominal aorta more than the thoracic aorta, with infra-renal abdominal aorta being the most common site [66,73,74,75]. Other reported complications of IgG4-related aortitis include aortic regurgitation, aortic stenosis, and aneurysmal rupture (estimated at 42% of cases) [1,76,77]. Mizushima et al. proposed diagnostic criteria for IgG4-related aortitis/peri-aortitis that required abnormal imaging findings consistent with aortitis, histological features, or an elevated IgG4 level, and an organ-specific IgG4-RD [78]. Characteristics of histological findings are described in Table 1b [62,78,79] and illustrated in Figure 2. Infiltration of the tunica media leads to aneurysm dilatation and rupture. No specific tests are available, but elevated sIL-2R, hypocomplementemia, ANA positivity, and rheumatoid factor positivity are often found in association [64,78,80]. Elevated CRP should prompt consideration of alternative diagnoses, including IgG4 [65]. Various imaging modalities, including ultrasound, CTA, MRA, and FDG-PET (Figure 7 and Figure 8), have been used to evaluate IgG4-R aortic disease. The most common CT findings are diffuse arterial wall thickening with homogeneous enhancement and a surrounding non-stenotic mass with irregular margins [74,81,82,83]. Vessel distribution classification is summarized in Table 4. Aneurysmal dilatation is found in only 7% [82], and stenoses/strictures are rare [66,82,83]. The incidence of an aneurysmal rupture is low in cases with severe aortic wall thickening and surrounding adhesions, but this does not apply to saccular aneurysms [84,85]. FDG-PET, in addition to CT or MRI, identifies active inflammation, guides biopsy site selection, and monitors treatment response [86,87,88,89]. Most patients respond well to moderate-dose steroids (0.5–6 mg/kg/day); higher doses are required for severe disease with multiple organ involvement. Caution should be exercised in large aneurysms prone to rupture [64,86] and in peri-aortitis, in which rapid thinning of the aortic wall is observed post-steroid treatment [73]; 20% of patients showed aortic dilatation post-steroid treatment [74]. A combination of steroids with steroid-sparing agents is superior to steroids alone in achieving remission [64,67]. The FDA approved inebilizumab, which targets and depletes CD19+ B-cells based on a recently published randomized controlled trial for the treatment of IgG4-related disease. Inebilizumab has also shown benefit in a phase 3 clinical trial, with an annualized flare rate of 0.14 (95% confidence interval, 0.06–0.31; *p* < 0.001) [87]. Benefits with MTX, MMF, AZA, leflunomide, CYC, and rituximab have been reported in conjunction with steroids [64,88,89,90,91]. Rituximab can also be utilized as a monotherapy or in steroid-refractory cases [66]. The standard treatment guideline for usual aneurysms dictates surgical intervention. Post-operative steroids may be administered in cases of persistent inflammation [73] (Figure 11 and Figure 12).

### 3.6. Relapsing Polychondritis (RP)

**Figure 13:**
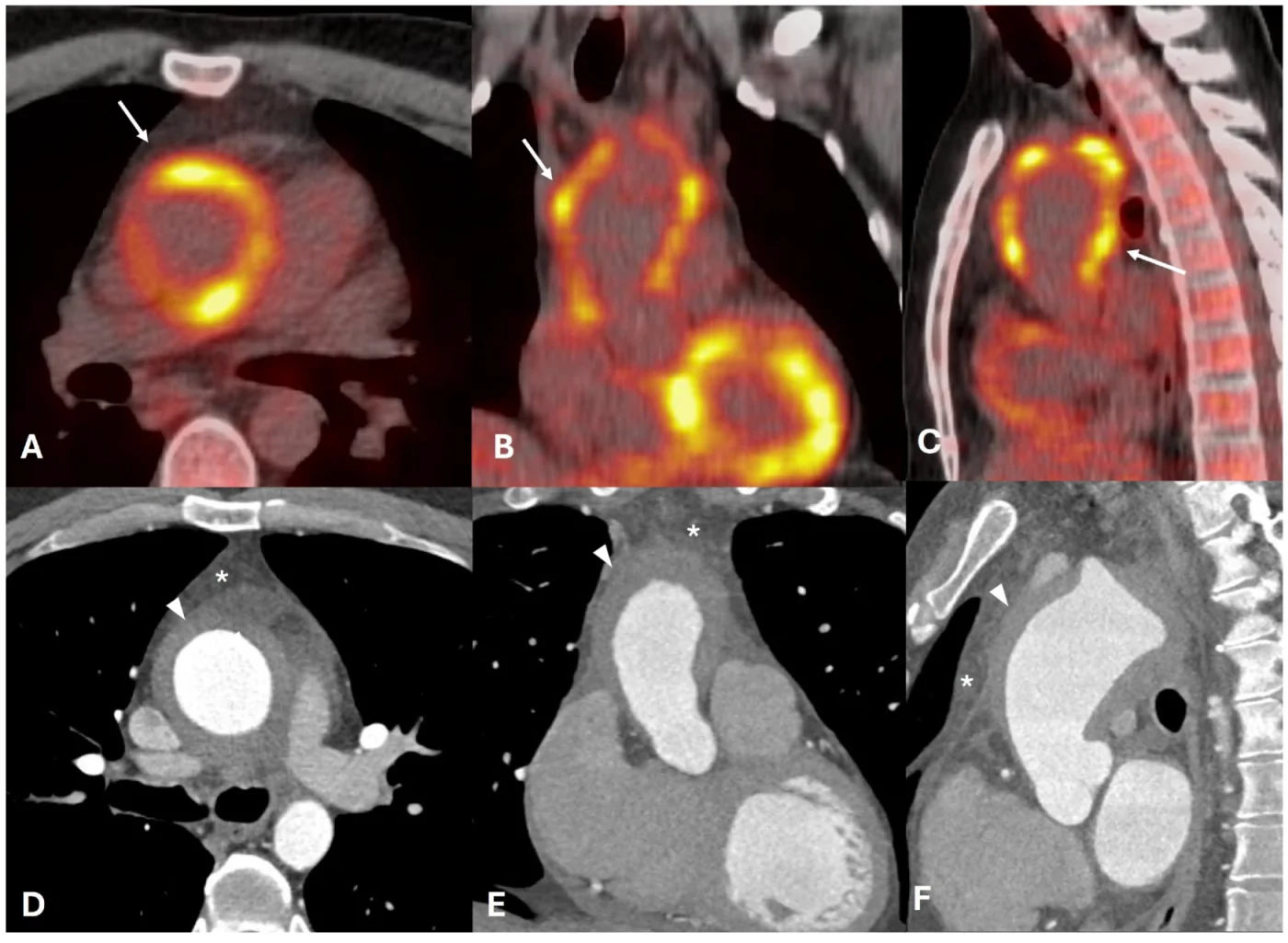
An example of known active relapsing polychondritis evaluated for chest pain. Axial (**A**), coronal (**B**) and sagittal (**C**) fused images of F-18 FDG PET/CT showing moderate intensity heterogeneous mural tracer activity throughout the ascending aorta involving the aortic root consistent with active aortitis (arrows). Axial (**D**), coronal (**E**) and sagittal (**F**) images of chest CTA showing concentric mural thickening to the ascending aorta (arrowheads) with surrounding infiltration to the mediastinal fat (*) corresponding to areas of abnormal tracer activity seen on PET/CT.

Cardiovascular complications are found in about 25% of RP cases and are the second-most common cause of death [92]. These include aortic regurgitation secondary to aortic root dilation, mitral regurgitation, atrioventricular block, myocarditis, pericarditis, and aneurysm of arteries, including the aorta [93,94]. In a retrospective study of 172 patients with RP, 11 patients (6%) had aortic involvement, with a median interval of 27 months from disease onset [95]. These included isolated thoracic aortitis, isolated abdominal aortitis, or a combination of both aortic aneurysm and aortic ectasia [95]. In a smaller retrospective cohort of 33 patients with relapsing polychondritis, the prevalence of aortic complications was also at 6% [96]. The major cause of death is a ruptured AAA [95]. Diagnoses were made by aortic CTA, MRA, or FDG-PET/CT (Figure 9) [95,97]. Of all RP cases with aortic involvement, the majority have isolated aortic vessel involvement, with the ascending aorta being the most common site; some have only aortic valve involvement, while others retain both aortic vascular and valve involvement [98]. The treatment goal is to control the inflammatory process and suppress the underlying immune-mediated destruction while minimizing the adverse effects of medications. This includes a stepwise approach of NSAIDs, dapsone, and colchicine in less severe cases, and corticosteroids or steroid-sparing agents (MTX, AZA, CYC, and MMF) in more severe cases [93]. Several biologics may be used in cases that are resistant to conventional therapies, but evidence is very limited [93,95]. In cases of refractory to conventional IST, infliximab, adalimumab, or tocilizumab have been given [95]. Tocilizumab was also reported to be effective in resolving symptoms and normalizing ESR and CRP levels [99,100]. Surgical interventions were required in some patients [95]. Relapse of aortic involvement was more common in patients with aortitis and abdominal aorta involvement [95]. Higher ESR levels and the presence of aortitis were associated with increased mortality [95]. Therefore, early and aggressive IST could prevent the progression of aortic complications [101] (Figure 13).

### 3.7. Cogan’s Syndrome

Cogan’s syndrome is a rare autoimmune disease characterized by ocular inflammation and vestibulo-auditory abnormalities, which occur predominantly in children and young adults. Systemic vasculitis can be seen in 10–13% of cases, mainly affecting large vessels indistinguishable from TA and medium vessels (polyarteritis nodosa-like) [102,103]. Aortitis occurs in up to 10% of cases and at any time between 2 weeks and 12 years after diagnosis of Cogan’s syndrome [4,104]. The transmural inflammation of the aorta seen on histopathology results in aortic aneurysm and aneurysmal dilatation, leading to rupture--one of the causes of death in Cogan’s syndrome [105,106]. The pathology usually affects the ascending aorta and arch but can be anywhere from the aortic root to the distal part of the aorta with or without aortic insufficiency, aneurysm, dissection, dilation, or stenosis/thrombus [102,103,105,107,108,109,110,111,112]. Multiple imaging modalities, such as ultrasound, CT/CTA, MRI/MRA, and angiography, have been utilized to diagnose the structural abnormalities of the aorta, but PET F18-FDG has gained popularity for aortitis diagnosis as it is also beneficial for evaluating treatment response during follow-ups, similar to its use in TA [113,114,115]. Systemic corticosteroids (1–1.5 mg/kg/day of prednisone) effectively control the disease. Reported immunosuppressive agents, including MTX, AZA, cyclosporin (CSA), and CYC, may be indicated in severe or refractory cases. Surgical management is indicated if symptomatic [102,104].

### 3.8. Clinically Isolated Aortitis

Clinically isolated aortitis is a poorly understood subgroup of patients with inflammatory changes on histopathology or imaging that involve only the aorta and are not associated with systemic clinical signs or symptoms. Histopathology will resemble that seen in GCA, with granulomatous inflammation and giant cells, though it may also include lymphoplasmacytic or mixed inflammatory phenotypes [116,117]. When these patients are followed, approximately 40–75% develop additional vascular lesions [118]. Thus, in this group, some experts recommend clinical observation rather than immunosuppressive treatment [119].

### 3.9. Rheumatoid Arthritis

Rheumatoid arthritis (RA) with extra-articular manifestations like aortic involvement is uncommon. Rheumatoid aortitis is associated with a more generalized rheumatoid vasculitis. Involvement can be seen in either the thoracic or abdominal aorta, with inflammation potentially present in all layers of the aortic wall [4]. Patients with RA exhibit heightened aortic inflammation, detectable through imaging modalities such as FDG-PET. This subclinical vasculitis may play a contributory role in the augmented cardiovascular disease risk observed in RA patients [120]. A case–control study by Shovman et al. in 2016 revealed an independent association between the aortic aneurysm and RA after adjustment for different factors, including age, gender, socio-economic status, and smoking status (OR 1.406, 95% CI 1.094–1.789; *p* = 0.006) [121]. Aortic stiffness is markedly increased in patients with RA compared to healthy controls, indicating that the aorta is particularly susceptible to the inflammatory processes associated with RA. Increased aortic stiffness and accelerated atherosclerosis are closely linked to adverse clinical outcomes, including cardiovascular risks, increased rates of hospitalization, and mortality [122]. Case reports and clinical studies have also documented granulomatous involvement of the aortic valve and the aorta in RA, a condition that can lead to aortic valve insufficiency and may require surgical intervention [123]. The management of aortitis in RA primarily targets the underlying inflammatory process. Corticosteroids are the first-line option for controlling acute inflammation. Long-term management necessitates the use of steroid-sparing agents, such as methotrexate or other disease-modifying antirheumatic drugs (DMARDs), to minimize corticosteroid adverse effects [124]. Anti-TNF-α therapy has been shown to reduce both aortic inflammation and stiffness in patients with RA, highlighting its potential role in managing aortitis. Surgical interventions, such as aortic valve replacement, may be required for significant aortic insufficiency. However, surgical or endovascular procedures should be postponed until the inflammatory disease is well-controlled to reduce the risk of complications [124].

### 3.10. Behçet’s Disease (BD)

**Figure 14:**
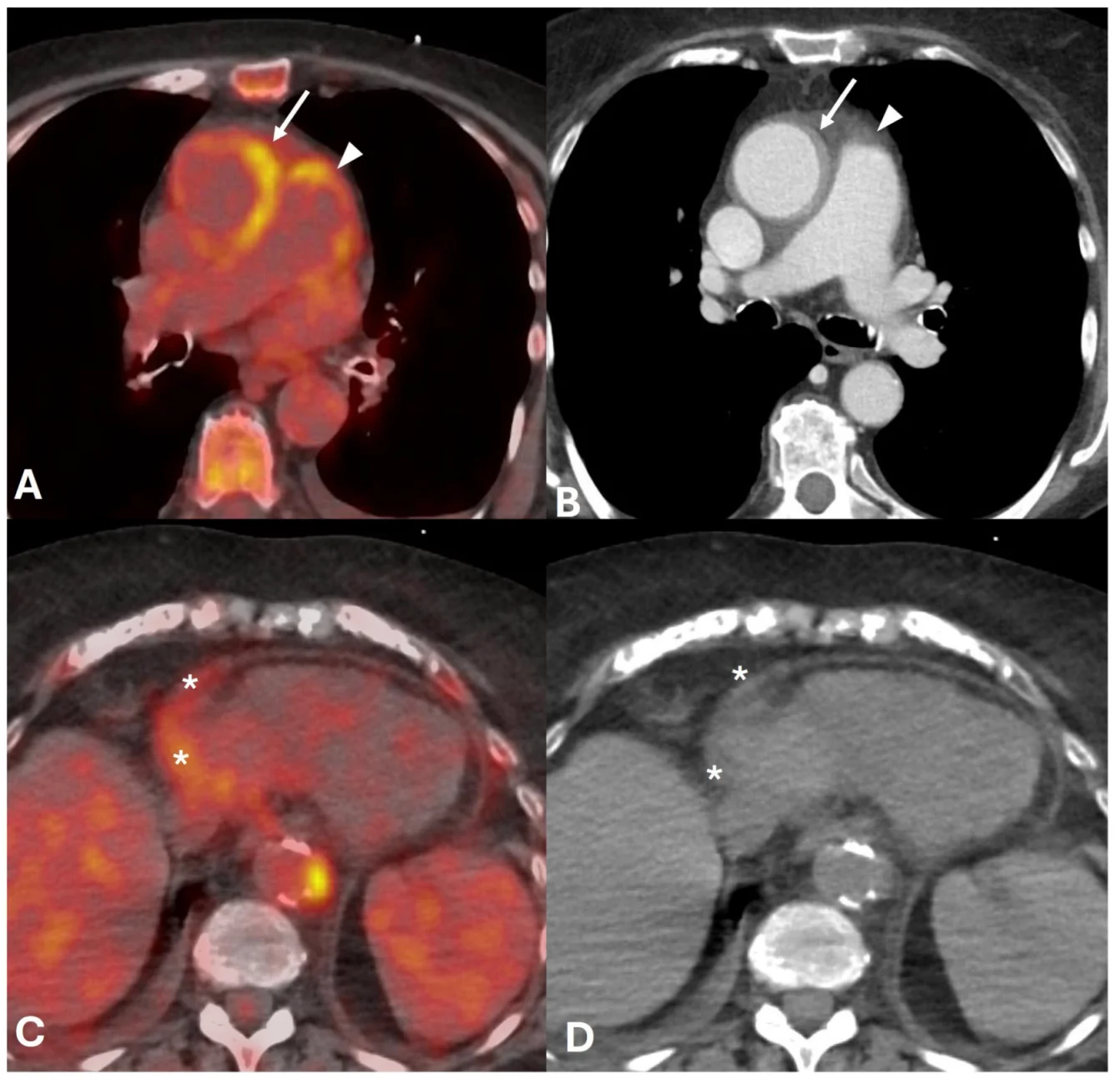
An example of known Bechets disease presenting with active vasculitis and pericarditis. Axial fused images of F-18 FDG PET/CT (**A**,**C**), axial image of CT chest with intravenous contrast (**B**) and axial image of CT chest without contrast (**D**) showing tracer avid concentric mural thickening to the ascending aorta (arrows) and to a lesser extent main pulmonary artery (arrowheads) as well as tracer avid pericardial thickening (*).

Vascular involvement is a common finding, occurring in up to 50% of patients with BD [125]. Arterial involvement is found in 2–18% of patients, usually involves the aorta and peripheral arteries, and more commonly results in aneurysm formation than stenosis or occlusion [126,127]. Aortitis can be associated with aortic regurgitation [128,129]. In a retrospective cohort study, an arterial aneurysm was found in 18% of patients with BD, all of whom were male [130]. Involvement of the pulmonary artery (44%), abdominal aorta (15%), and ascending aorta (7%) was among the most common, and 44% had multiple aneurysms [130]. In another study, involvement of the ascending aorta, aortic arch, descending aorta, and abdominal aorta was found in 28%, 4%, 12%, and 30% of cases with arterial involvement, respectively [131]. The angiographic features seen in the aorta were aneurysm (46%), dilatation (20%), and stenosis (6%) [131]. Imaging modalities used to detect these abnormalities include PET, CTA, MRA, and invasive angiography (Figure 10) [22]. Compared with TA, patients with BD and arterial involvement had more constitutional symptoms and higher inflammatory marker levels, but fewer vascular symptoms [131]. Aneurysm formation was more common in BD, while stenosis and occlusion were more common in TA [131]. BD was more commonly associated with vascular complications, including aortic regurgitation, arterial dissection, and arterial rupture, and was associated with higher vascular interventions [131]. For aortic involvement, medical treatment with high-dose GCs and CYC is warranted before surgical intervention [132]. For the treatment of aortic pseudoaneurysms requiring intervention, pre- and postoperative immunosuppressive agents, in addition to endovascular stent grafting or surgical correction, resulted in favorable outcomes [133,134]. In a small cohort of patients with severe vascular involvement, including aortitis and aortic dissection, infliximab was shown to be effective in rapidly inducing and maintaining remission of vascular inflammation [135] (Figure 14).

### 3.11. Spondyloarthropathy (SpA)

An important cardiovascular manifestation of ankylosing spondylitis (AS) is aortitis, which involves the aortic root and ascending aorta and leads to valvular insufficiency, especially in long-standing disease [6,136]. The majority of patients are asymptomatic. Aortitis gradually causes dilatation of the aorta and slow progression of aortic insufficiency; however, acute aortic insufficiency with rapid worsening of cardiac function is not impossible [136]. The lone aortic regurgitation is characterized by annulus dilatation, thickening, and inward rolling of aortic cusps, in which the dilation can involve the ascending aorta. Aortic root abnormalities (thickening, increased stiffness, and dilatation) may be seen in AS [137]. Diminished aortic elasticity does not correlate with the duration of AS [138]. Thoracic or abdominal aneurysm dilatation has been described as rare [136,139,140]. An echocardiogram has been used to detect early aortic root involvement in AS and reactive arthritis syndrome, while PET/CT has been utilized to evaluate inflammatory activity [141,142,143]. Aortic involvement in other forms of SpA is rare. Psoriatic arthritis/psoriasis is known to be associated with atherosclerotic disease, with increased aortic stiffness [144,145] and has a higher risk of aortic aneurysm [146]. Medical treatment of aortitis in AS is usually effective; Sulfasalazine (SSZ), prednisone, CYC, or TNFα inhibitor use has been previously reported [147,148,149,150], but some patients may require surgical intervention in addition to medication [150,151,152].

### 3.12. Systemic Lupus Erythematosus (SLE)

SLE-associated aortic disease is rare. Most aortic aneurysms are degenerative and not inflammatory [4]. Aortic aneurysms are more common in SLE than in the general population and occur at a younger age [153,154]. Most cases of lupus-associated aortitis have been reported in association with aortic aneurysms or aortic dissection, often identified post-mortem or during surgical intervention [153,155,156,157]. Over 70% of SLE aortitis cases involve the thoracic aorta. An echocardiogram, either transesophageal or transthoracic, is useful for assessing aneurysm formation, dissection, and signs of inflammation, such as mural thickening [22]. However, CTA and MRA can provide a better comprehensive assessment of the entire aorta [22]. Recently, the FDG-PET has proven to be a valuable diagnostic tool for detecting aortitis in SLE and for monitoring therapeutic response in these patients [158]. The treatment of aortitis in SLE remains inadequately defined due to its rarity. Glucocorticoids are typically the first-line therapy for managing lupus-related aortitis, with moderate-dose glucocorticoids shown to effectively resolve symptoms and laboratory abnormalities, as evidenced by a case report where such treatment led to clinical improvement [158,159]. Aortitis associated with catastrophic antiphospholipid syndrome (CAPS) necessitates a broader immunosuppressive approach and anticoagulation. This may include a combination of anticoagulants, corticosteroids, hydroxychloroquine, intravenous immunoglobulins (IVIg), plasma exchange, and rituximab, as demonstrated in a case of both SLE and CAPS that responded favorably to this multifaceted approach [160].

### 3.13. Antiphospholipid Syndrome (APS)

APS is often associated with other autoimmune disorders, such as SLE, but it can also be present in isolation [161]. Cardiovascular involvement can be found in primary antiphospholipid syndrome but is more common in APS secondary to other autoimmune disorders, such as SLE [161,162]. These include heart valve vegetation, thickening, and dysfunction [163,164]. Primary APS is rarely associated with chronic PA [165]. It is more common to see aortic involvement, such as aortitis and abdominal aortic aneurysm (AAA), in APS cases associated with SLE, usually in young female patients [160,166,167]. In a study by Duftner et al., the presence of antiphospholipid antibodies was an independent predictor of AAA disease progression in patients with AAA [168]. However, it was not associated with the presence or the volume of intra-aneurysmal thrombus [168]. The main treatment goals of antiphospholipid syndrome are to prevent thrombosis and pregnancy complications. These include the use of vitamin K antagonist, heparin, and/or low-dose aspirin [169].

### 3.14. Sjogren Syndrome (SS)

A link between Sjögren’s syndrome (SS) and aortic pathology, particularly aortic aneurysm and aortic dissection, has been reported. A nationwide, population-based cohort study in Taiwan found that patients with SS face a significantly more than threefold increased risk of developing aortic aneurysm or dissection compared to the general population. This finding suggests that the systemic inflammation and immune dysregulation inherent to SS may contribute to vascular changes that predispose individuals to aortic complications [170]. Furthermore, SS has been implicated in the functional impairment of the arterial wall, which may facilitate early atherosclerotic changes. This impairment is thought to arise from a combination of chronic inflammation and immune-mediated damage, impacting endothelial function and vascular smooth muscle cells [171]. The elevated cytokine levels and increased expression of adhesion molecules may exacerbate vascular damage and promote the development of atherosclerosis, a risk factor for the formation of aortic aneurysms and dissections [171]. The management of SS with systemic involvement, such as aortic pathology, involves the use of GCs and immunosuppressive agents. GCs are frequently employed for their rapid anti-inflammatory effects, while immunosuppressive drugs, including cyclophosphamide (CYC), AZA, or MMF, may be considered for long-term management to control disease activity and mitigate progression. Rituximab demonstrated potential in addressing systemic manifestations of SS; however, its use remains largely off-label and is based on limited clinical evidence [172].

### 3.15. Systemic Sclerosis (SSc)

Vascular abnormalities of SSc are notable in the microvascular system. Large vessel involvement in SSc, including the aorta, is uncommon [173]. Aortic involvement is mainly characterized by progressive aortic stiffness due to chronic-inflammation-mediated loss of elasticity and fibrotic remodeling of the aortic wall, [174,175,176,177], and the atherosclerotic process [178]. A few cases reported aneurysm formation or dissection; the aneurysms are presumed to be caused by vasculopathy of the vasa vasorum of the aorta, causing vessel wall weakness [173,179,180,181].

### 3.16. VEXAS Syndrome

VEXAS (Vacuoles, E1-enzyme, X-linked, autoinflammatory, somatic) syndrome is a recently identified clinical entity first described by Beck et al. in 2020 [182]. It is caused by an acquired mutation in the bone marrow that preferentially affects the myeloid lineage, leading to widespread inflammatory manifestations and hematologic abnormalities, including macrocytosis and cytopenias. The majority of afflicted patients are men because the gene is X-linked, though rare cases of female patients have been reported, with an estimated prevalence in men over the age of 50 at 1 in 4269 [183]. Many patients with this diagnosis had previously been classified with a range of other defined RD’s, including Cogan’s syndrome, BD, and GCA [182]. Current literature suggests that VEXAS syndrome induces a variable vessel vasculitis, with up to 1/5 of patients having vessel inflammation, though the majority of these will have small to medium vessel cutaneous disease [184]. Aortitis and other large-vessel vasculitis are an recognized manifestations. This has been identified in case reports using CTA and MRA [185,186]. Little information is known about the histopathology of VEXAS syndrome, and no characteristic pattern has been established; temporal artery biopsy is usually unrevealing, with a positive biopsy showing focal neutrophilic-predominant inflammation in the intima reported in only 1 of 18 biopsied patients in a recent cohort [184].

### 3.17. Sarcoidosis

Aortic involvement in sarcoidosis is rare but well recognized. The rare incidence of aortitis can result in aortic aneurysm/dilation, aortic dissection, stenosis, or pseudoaneurysm with aorto-esophageal fistula [187,188,189]. Pathology has reportedly been found in any section of the aorta [187,188,190,191]. Medial necrosis and loss of elastic lamina (loss of elasticity) due to granulomatous inflammation result in an aneurysm, aneurysm expansion, and possibly rupture due to the fragility of the aneurysmal wall [115,192,193,194]. Histopathology of aortic walls shows classic non-caseating granulomas similar to other organs, but non-specific features like atherosclerotic changes and aortic calcification due to chronic inflammation were also identified [115,190,195,196,197]. CTA/MRA and angiogram have been beneficial in visualizing structural abnormalities, and PET F18-FDG has been applied for functional evaluation [188,198,199]. Like sarcoidosis in other organs, the mainstay of treatment is corticosteroids (1–2 mg/kg/day or prednisone equivalent). Other steroid-sparing agents may be indicated depending on organ involvement and clinical response [187,188]. Surgery is indicated for aneurysms and dissections [6]. A summary of Key points for the differential diagnosis of aortitis/periaortitis among rheumatic diseases presented in Table 5.

## 4. Investigation, Diagnostic, and Monitoring Modalities

### 4.1. Imaging Modalities

Ultrasound, CT, MRI, and PET have been widely used to detect aortitis, the inflammatory and structural changes in the aorta, replacing conventional angiography and direct gross tissue and histological examinations [22]. The EULAR 2023 update has recommended ultrasound as the first-line imaging test in all patients with suspected GCA, and axillary arteries as the standard examination. FDG-PET or MRI can be used as alternatives to ultrasound for assessing cranial and extracranial arteries. For TA, MRI is the preferred imaging modality; FDG-PET, CT, or ultrasound are alternatives [200]. Conventional angiography detects only luminal abnormalities, not the vessel wall [200,201,202], and is reserved for cases undiagnosed by non-invasive imaging modalities or those requiring revascularization [6]. Ultrasound, including echocardiography, has been beneficial in detecting aortic root and aortic valve abnormalities, mural thickening, AAA, and dissection [6,201]. The addition of Doppler helps assess impediments to blood flow. Portability and the lack of ionizing radiation are advantages, but diagnostic accuracy is operator-dependent [6,202]. CTA is widely available; it can detect inflammation (mural wall thickening at >2–3 mm), enhancement, stenosis/occlusion, and surrounding structures of the whole aorta, including extent and sequelae of aortitis, with three-dimensional reconstruction [22]. It allows rapid exclusion of aortic pathologies [6]. Perfusion CT is an imaging tool, in addition to morphological CT, for monitoring disease activity in response to immunosuppressive therapy, particularly in settings where inflammatory markers are initially negative [203]. However, among these modalities, CTA is the most favored diagnostic tool because it can quantify disease severity and provide adequate images for follow-up [17]. The main disadvantages of CT are contrast and radiation exposure, temporal resolution, and non-portability. MRA is similar to CTA but can also detect subtle edema with the advantage of no radiation exposure. However, it requires a longer image acquisition time, is not appropriate for unstable patients, and is contraindicated in patients with certain devices or implants [22,201]. PET imaging uses fluorine-18-fluorodeoxyglucose (18F-FDG) to assess inflammation in aortitis [204]. Co-registration of 18F-FDG PET with CT or MRI enhances diagnostic yield [205]. PET-CT has an advantage over other imaging modalities, as it can identify early inflammatory changes with a sensitivity of 76% and a specificity of 93% [206]. However, it may not be able to distinguish atherosclerosis from vasculitic inflammation. High-intensity, diffuse uptake patterns and lesion localization can assist in distinguishing LVV from atherosclerosis [2,206] and require other supportive clinical signs and symptoms to meet the criteria for each rheumatic disease [202]. Atherosclerotic disease also tends to be eccentric rather than concentric and has an increased incidence of calcification [207]. Not as widely available as PET-CT, PET-MRI with a vasculitic activity scoring system (sensitivity 77%, specificity 88%) can distinguish active from inactive LVV [208] (Table 6).

### 4.2. Laboratory Work-Up

Aortitis is progressive and may be silent or only associated with non-specific symptoms. Localizing symptoms develops only when a complication occurs. Early detection is pivotal for identifying and monitoring disease activity and for directing early therapy [209]. Aortic valve insufficiency, renal or mesenteric occlusion, decreased perfusion to the extremities, or aortic aneurysms without typical risk factors in patients with rheumatic disease should raise concern for aortitis [210]. Therefore, screening for aortitis in the early stage of RD is challenging. Serologic testing can provide clues to an underlying etiology, but it is frequently unable to provide a definitive diagnosis. An IgG4 level of more than 5 times the upper limit of normal may indicate, but is not sufficient for a diagnosis of IgG4-related disease. ADDIN EN.CITE [210] ANCA titers can be obtained to delineate patients with possible ANCA-associated vasculitis. In males > 40 years of age with suspicious hematologic features, genetic testing for UBA1 mutations can establish the diagnosis (e.g., VEXAS syndrome). Unfortunately, the majority of patients with aortitis are diagnosed through clinical features alone, given the logistical difficulties and risk of biopsying aortic tissue, though histopathology can be helpful in the event it is obtained through surgery.

### 4.3. Conventional and Emerging Laboratory Biomarkers

PA, like aortitis, has been found to be associated with rheumatic disease. ESR, CRP, and leukocyte count are often used for disease monitoring; however, these may not always correlate with disease activity, especially with PA [211,212]. A study by Blanken et al. demonstrated that immunosuppressive treatment in RA significantly reduced ESR, CRP, and inflammation of the abdominal aorta, but not of the thoracic aorta, as detected on FDG-PET after 6 months [203]. The current comprehensive literature includes only those common rheumatic disease-related aortopathies. About 40% of TA patients and 22% of GCA patients had normal acute-phase reactant levels during active disease, necessitating a more sensitive diagnostic tool [213]. IgG4 levels are variable in patients with IgG4-RD, and definitive diagnosis may require histopathology [214,215,216,217]. Acute-phase reactants (ESR, CRP) are nonspecific and do not correlate with disease activity [218,219]. Several biomarkers have been implicated in the activity of TA, including IL-6,IL-17, and pentraxin-3 (PTX3) [17]. IL-6 has been shown to correlate with disease activity in both TA and GCA consistently [220,221,222,223]. Compared with acute-phase reactants, IL-6 is a more sensitive marker of active disease and disease flares on follow-up [220,224,225], though it may be affected by treatment with IL-6 inhibitors such as tocilizumab [226]. FGD-PET uptake correlates with ESR, CRP, and IL-6 [37,227,228]. Other biomarkers, IL-8, IL-18, and MMP-9, have been investigated in TA and GCA with inconclusive results [229,230,231]. Several other serum markers, including GM-CSF, IL-10, IL-12, IL-23, circulating endothelial cells, cellular adhesion molecules, platelet and coagulation activity markers, and smooth muscle myosin, have also been tested and found to be non-beneficial [230,232]. A meta-analysis has suggested that PTX3, an independent inflammatory marker unrelated to ESR and CRP, may be a superior marker for assessing disease activity relative to CRP, with a sensitivity of 78% and specificity of 85% [17]. PTX3 has been suggested as a more specific marker of arterial inflammation, making it an appealing choice for aortic involvement [233]. Its levels in active TA patients are higher not only in healthy patients but also in those with inactive TA disease or infections [234]. Despite these encouraging reports, a follow-up study in a bigger cohort of patients did not find any correlation between TA disease activity and PTX3 levels [235]. PTX3 has had conflicting reports: an early study showed higher specificity and predictive value in TA, but a later, larger study failed to confirm these results [231,233,234]. A recent study by Carmona et al. showed that the HLA class II DRB1 gene is associated with GCA, while the HLA Class I MICA shows an association with TA [236]. Mendelian randomization analysis showed a significant association between BD and AAA [237]. Three hub genes (CD2, CD247, CCR7) were identified by bioinformatics analyses as potential biomarkers for AAA in BD [237]. Another study suggested that higher serum matrix metalloproteinase 2 (MMP-2) levels were associated with vascular involvement, particularly aneurysmal pathology [238] (Table 7).

### 4.4. Management

Immunosuppressive therapy is the mainstay of treatment in aortitis secondary to rheumatic disease. A multidisciplinary team, which includes a rheumatologist, cardiologist, and vascular surgeon, should be considered for the care of patients [6]. Systemic glucocorticoids are considered in most of the rheumatic conditions at various initial dosages to achieve clinical remission before tapering [6]. Short-term and long-term side effects of steroids should be monitored. Steroid-sparing agents may be indicated as adjunctive therapy to allow tapering of steroids or in recalcitrant/relapsing cases and as first-line therapy in many conditions [201,239]. The growing role of novel targeted agents, e.g., IL-6 and TNF-α inhibitors, is directing medical treatment towards targeted therapies, such as biological DMARDs. The choice of targeted agent is guided by the underlying disease: interleukin-6 receptor inhibition with tocilizumab is a first-line and steroid-sparing option in giant cell arteritis and is used in relapsing or refractory Takayasu arteritis, whereas tumour necrosis factor-α inhibitors are preferred in Takayasu arteritis, Behçet disease and ankylosing spondylitis; infliximab is effective for severe vascular Behçet disease, and glucocorticoids with cyclophosphamide are used for aortic involvement in systemic vasculitides such as ANCA-associated vasculitis. It is important to note that medical treatment does not alter aneurysmal changes, and chronic inflammation does not predict the progression or rupture of aneurysms [239,240]. Asymptomatic thoracic or abdominal aneurysms should be monitored periodically for any surgical indication [6,31,241]. Preferably, inflammation of the aorta should be suppressed medically pre- and post-operation, and ideally, clinical remission should be achieved before an elective aortic vascular repair [210,242]. For symptomatic aortic occlusive disease secondary to any rheumatic conditions, revascularization and surgical bypass graft are available options.

### 4.5. Surgical Considerations

**Figure 15:**
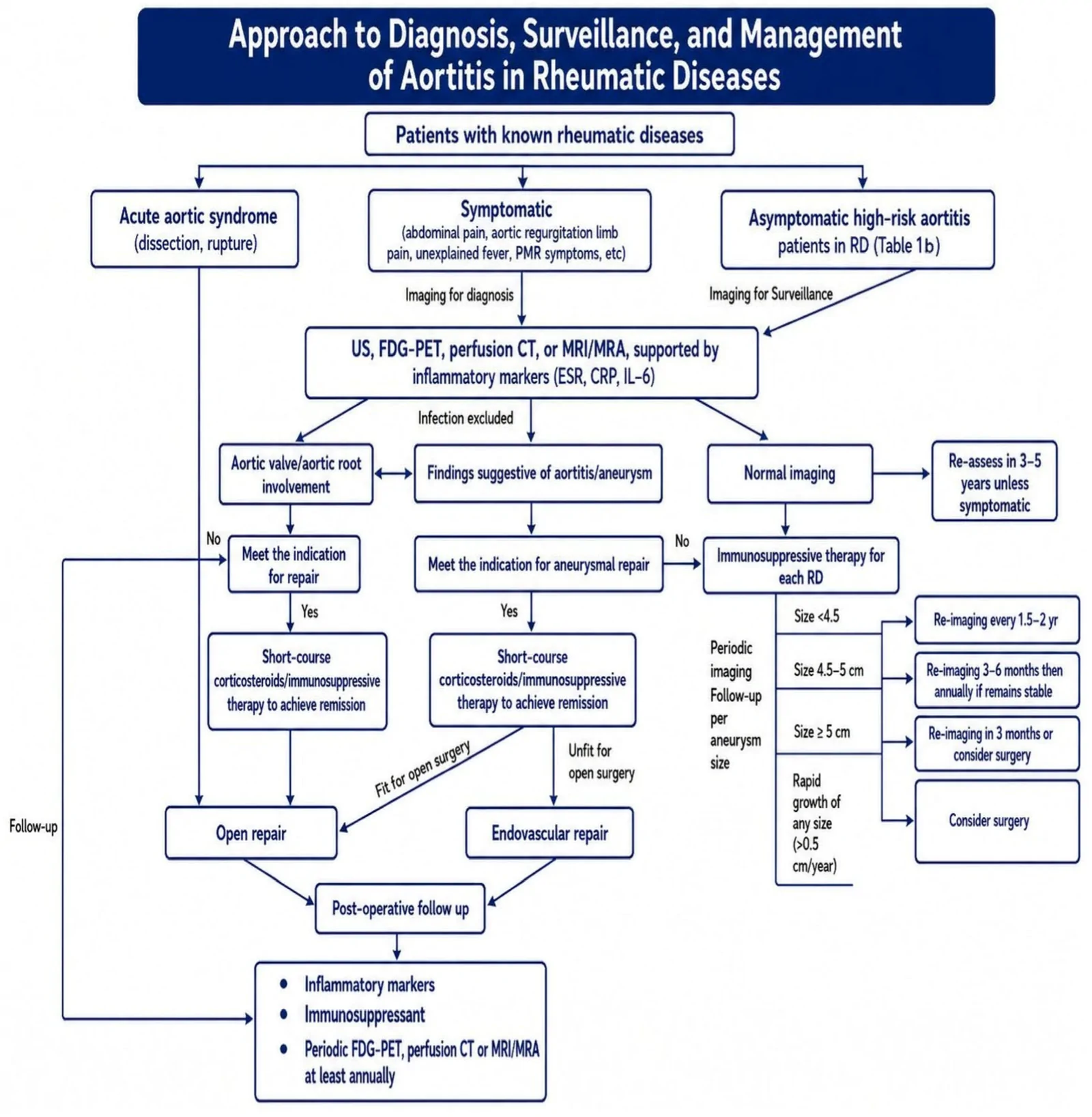
Proposed management algorithm and periodic imaging in rheumatic disease (RD) patients with aortic involvement.

Aortitis-related rheumatic disease contains no guideline management. ESC guidelines for the management of aortic disease regarding aneurysmal aortitis did not define the threshold at which surgery is recommended, and the risk of rupture is unknown [243]. Therefore, the diameter criteria for repairing asymptomatic inflammatory aneurysms follow the same guidelines as non-inflammatory aneurysms [244,245]. Although no clear evidence supports surgical intervention at a specific aortic diameter, intervention at a lower aortic diameter can be considered in aortitis because of the high incidence of acute complications [246]. Similarly, indications for endovascular and conventional surgical management of arterial occlusion in RD are assessed in the same way as for non-inflammatory causes [210]. Open surgery remains the standard of treatment in aortic aneurysms associated with aortitis, although endovascular intervention has gained popularity with reported success [6]. The decision should be based on the aneurysm’s location, size, and shape, as well as the patient’s past medical history and the surgeon’s expertise [247]. In elective surgery, administering corticosteroid and immunosuppressive agents preoperatively to suppress inflammation was associated with fewer postoperative complications [248,249]. A multidisciplinary care team should determine the timing of the intervention [250]. For emergent cases requiring immediate intervention, corticosteroids, conventional DMARDs, and biological DMARDs may be necessary peri- and post-operatively, with assistance from a multidisciplinary team, including rheumatologists, to reduce complications and recurrences [124,247]. The recent guideline recommends EVAR for inflammatory abdominal aneurysms with appropriate anatomy as the first-line treatment [244]. EVAR may be more beneficial in patients with active inflammation, as open repair is associated with pseudoaneurysms at the suture line [251,252]. The increased risk of iatrogenic injury during open repair poses a higher risk than a degenerative aneurysm, especially in cases where PA with retroperitoneal fibrosis occurs, e.g., IgG-4-related aortopathy. As a result, the intra- and post-op morbidity and mortality are reportedly higher (6–11%) compared to non-inflammatory abdominal aneurysms (non-IAA) [253]. Therefore, EVAR can be considered in such cases [254]. A study by Caradu et al. reported no differences in 1- and 2-year mortality between open surgery and EVAR in the inflammatory abdominal aorta (not specified to a specific RD), but EVAR was associated with greater post-operative inflammatory progression in IAA [255]. This may indicate that to mitigate post-operative morbidity (second procedure following complications) and mortality, open surgery should be kept at a minimum in these cases, and surgeons should opt for EVAR and continue post-op treatment with immunosuppressants to reduce the progression of inflammation. However, no data is available to compare which modality is preferred [256]. For inflammatory thoracic aorta, not specified to a specific RD, once the site of inflammation is respected, the recurrence is very low [257]. The introduction of TEVAR is safer and more feasible with anti-inflammatory treatment. In GCA, the development of aortic dissection in aortitis is attributed to delayed corticosteroid treatment from symptom onset to diagnosis [258]. Disease progression in the distal aorta is characteristic of GCA (11-fold compared to the control group [118,119,259]), with prior surgical intervention and progressive dilation of the weakened, inflamed distal aortic wall, thereby decreasing the feasibility of EVAR [260]. Post-operative immunosuppressants are recommended if there is evidence of inflammation beyond the resected segment [155]. This can be applied to RD, which causes systemic inflammation, and the degree of aortic inflammation may be more unpredictable. While GCA may require reintervention due to distal aortic progression, TA did not, which may reflect occlusive disease progression [259]. Revascularization, when inflammation has subsided, is highly recommended [261,262]. The rate of restenosis of angioplasty or stenting is higher compared to open surgery in TA [261,263,264,265]. Chronic inflammation caused by stent grafts can also lead to aortic complications. However, it can also lead to aortic wall weakness, as can the inflammation itself. Therefore, controlling inflammation with optimal immunosuppressive agents and doses is of utmost importance. Revascularization for TA, whether surgical or endovascular, has been shown to be safe and is associated with low morbidity and mortality rates [23]. Conventional bypass grafts typically achieve optimal long-term outcomes for occlusive disease [23]. Additionally, percutaneous transluminal angioplasty has yielded favorable results, particularly for short lesions [23]. About 20% of TA patients require surgery secondary to sclerotic vessels, not amenable to PCI, aneurysms with risk of rupture, or severe aortic regurgitation [242]. Open surgery is best performed when inflammation is controlled to prevent valve and graft detachment caused by tissue friability. The hybrid technique (open surgery plus endovascular repair) has been proposed for vascular reconstruction in TA. Open surgery and endovascular treatment are both effective options for treating aortic pseudoaneurysms [249,266]. The aortic valve can be involved, but the long-term durability of the aortic repair remains elusive [267]. Glucocorticoids pre-operatively to achieve remission in TA reduce surgical revision rates and are recommended for large-vessel GCA and aortitis [261]. A study by Caterson et al. demonstrated that most thoracic aortitis surgeries were performed electively, thereby allowing pre-operative medical management [268,269,270]. A Summary of the management algorithm is presented in Figure 15.

### 4.6. Novel Therapies

Studies on several novel treatments, including anti-cytotoxic T-lymphocyte antigen 4, Jak inhibitors, anti-IL-1, anti-IL17, anti-IL 12/23, anti-IL-23, anti-IL6, and other monoclonal antibodies, especially for TAK and GCA, are ongoing [2]. TNF-α antagonists and tocilizumab have shown a 93% improvement rate in DMARD-refractory Takayasu arteritis at 12 months, encompassing both complete and partial responders, while 75% of patients achieved a complete response with a favorable safety profile. Furthermore, TNF-α antagonists and tocilizumab appear to possess similar efficacy and tolerability, according to a multicenter retrospective study conducted on a French population by Mekinian and co-authors [18]. Baricitinib is a Janus kinase inhibitor that has the potential for treating vascular BD refractory to conventional and/or biological treatments [271]. In one small study, it was able to induce complete remission in 77% of such cases within 3 months without any serious adverse events reported [271]. Tocilizumab, an IL-6 receptor antagonist, may be another alternative for the treatment of refractory disease [272]. Several novel biologics, including TNF inhibitors, tocilizumab, anakinra, rituximab, abatacept, and tofacitinib, have shown promising results in cases of relapsing polychondritis [273,274]. However, none of these studies included cases with aortic involvement. Prospective randomized controlled trials with long-term monitoring are needed to assess the efficacy and safety of these novel biologics, although feasibility is a concern given the disease’s rarity. Novel approaches to target immunosuppressive therapy have emerged in recent years for the treatment of ARD and to achieve sustained remission. This has the potential to be used in the treatment of AAV and, hopefully, to extend to large-vessel vasculitis. Cellular- and antigen-specific therapies that have gained significant attention in the field of rheumatology include chimeric antigen receptor (CAR) T cells [275]. These are cytotoxic T cells isolated from the individual patient, genetically engineered to express a receptor that targets a specific antigen, expanded in culture, and then infused back into the patient. The most commonly approved CAR T-cell therapy on the market as of 2024 is CD19 CAR T cells. These CAR T cells seek out and destroy CD19-expressing B cells. Another off-the-shelf targeted therapy under consideration in ARDs that has great potential in vasculitis is a bispecific autoantigen T cell engager (BiAATE) [276]. One end of the BiAATE is a traditional anti-CD3 antibody that engages cytotoxic T cells. This portion is connected to a linker that attaches to the autoantigen, or a portion of it, of choice. The premise is that autoreactive B cells that recognize the autoantigen will be linked to cytotoxic T cells and brought into close proximity for cell death and elimination.

### 4.7. Monitoring, Follow-Up, and Surveillance

There has been no optimal method to screen for aortitis. Monitoring of asymptomatic aortic involvement should be individualized. Assessment of inflammation with ultrasound, CTA, and MRI is beneficial in detecting subclinical aortitis [277,278]. Although imaging is not routinely recommended for follow-up, ultrasound, FDG-PET, or MRI may be used to assess vascular abnormalities in LVV patients with suspected relapses, particularly when laboratory markers of inflammation are unreliable. CTA, MRA, or ultrasound [200]. 18F-FDG-PET-CT or MRI, used not only for diagnosis, but can also be used to assess disease activity and long-term monitoring of structural damage, particularly at sites of preceding vascular inflammation. and monitor response to treatment, especially in large-vessel vasculitis or rheumatic diseases with aortitis [200,204,227,239,279]. Although a routine follow-up with PET or MRI has no proven benefits in BD and RP, it can help detect large-vessel disease during relapses [38,40]. More literature on TA and GCA is more extensive and serves as a reference for other RDs with aortic involvement. Clinical symptoms remain the primary focus of monitoring patients with TA [280]. A comprehensive search, not limited to the presenting signs or symptoms, may be needed, as many cases involve multiple vascular sites [127,281,282]. Unexplained fever or elevated inflammatory markers in RD should trigger a search for occult vascular involvement [250]. Imaging techniques can provide valuable insights into disease progression and help detect subtle inflammation in arterial structures. The use of 18F-FDG imaging is supported by clinical findings from previous research by Karapolat et al., which showed its effectiveness in detecting subtle inflammation within large vessels [280]. In addition, current follow-up protocols for CTA recommend monitoring the aorta and its branches for disease progression at 6- to 12-month intervals [17]. Given the considerable morbidity and mortality associated with aortic aneurysms, the aorta should be evaluated routinely during the follow-up of patients with GCA. Aortic aneurysms may occur in the early disease process but can also occur more often several years after the diagnosis, occasionally after corticosteroid therapy discontinuation. Disease activity is usually resolved after corticosteroid treatment, but residual contrast enhancement from CT may still be observed. In a study by Marie et al., the majority of LVV patients with aortic abnormalities were symptomatic; half of them resolved or improved with vasculitis treatment, while the rest remained stable, and only a minority (3%) deteriorated [283]. Despite apparent clinical remission, up to 63–84% of scans exhibit persistent uptake, according to a study by Camellino and co-authors [30]. However, after 1 year of corticosteroid treatment, contrast enhancement resolved in 93.75% of cases in GCA patients [30]. Close long-term follow-up is recommended regardless of the treatment approach [284]. Imaging studies are crucial during follow-up, including FDG-PET-CT, perfusion CT, MRI, and MRA [284]. Ongoing surveillance with imaging and close follow-up for disease activity monitoring using clinical symptoms, serologies, and imaging is required, as perianeurysmal inflammation does not fully subside postoperatively and may result in complications (periaortic fibrosis, anastomosis-site pseudoaneurysm) [285]. There is a lack of evidence-based guidelines on the indications, frequency, and modalities of screening for aortic involvement [286]. However, it is not irrational to follow the standard guideline for surveillance imaging of aortic aneurysms, or even earlier, given inflammatory pathogenesis, which may accelerate aneurysmal expansion [22].

### 4.8. Risk Stratification

F-sodium fluoride (F-NaF) in PET radiotracer correlates with aortic degradation [287]. Forsythe et al. (SoFIA3 study) demonstrated markers of aortic degradation in aortopathy to predict aortic expansion. Individuals with the highest uptake of 18F-NaF in most diseased aneurysmal segments also had the fastest rate of aortic growth and rupture or repair [288]. Current guidelines recommend surveillance of the aorta for risk of dilation with the ultrasound in the abdominal aorta and preferably CT or MRI in thoracic aorta involvement. Routine molecular F-FDG PET use for aortitis has been increasing [209]. The European Alliance of Associations for Rheumatology (EULAR) recommends MRA as the first-choice imaging modality to investigate mural inflammation or luminal changes and confirm the diagnosis of TA. FDG-PET/CT and ultrasound are alternative imaging modalities, but the latter is of limited value for the assessment of the thoracic aorta [200]. There is no specific data for risk stratification for each rheumatic disease with aortopathy. However, there have been studies on inflammatory aortic aneurysms (IAA). A multicenter study by Lindblad et al. demonstrated that the risk of rupture of IAA is lower than that of degenerative aneurysms [289].

## 5. Future Directions and Research Gaps

Validated diagnostic criteria, specific treatment endpoints, and disease activity monitoring for each rheumatic disease with aortic involvement require further investigation [124]. Targeted treatment modalities based on the pathogenesis of each RD, including inflammatory cytokines and antigen-activation pathways, are still being developed; long-term safety and efficacy should be evaluated [124]. The new PET tracers will provide a more specific interrogation of aortic inflammation and thus enable early detection in aortic involvement surveillance [209]. A head-to-head trial comparing open surgery and EVAR for aortic involvement in RD is an area for future investigation.

## 6. Role of AI in the Identification of Aortopathies

Studies have been developed to assist in the diagnosis of aortopathies such as aortitis, aneurysm, and dissection. A study by Duff et al. utilized radiomic analysis of FDG-PET images to detect active aortitis in suspected cases or patients with systemic inflammatory responses, with good diagnostic performance [290]. Hata et al. performed a study with an AI model that allowed the detection of aortic dissection by CT imaging, with an average sensitivity and specificity of 91.8% and 88.2%, respectively [291]. A study by Huang et al. used a different model to detect aortic dissection, which is classified as type A or type B. They achieved sensitivity and specificity of 95.5% and 98.6% for type A dissection, 79.3% and 94.1% for type B dissection, and 94.5% and 94.1% for no dissection, respectively [292]. Another comprehensive review by Huang et al. concluded several models from various studies for the screening and diagnosis of aortic aneurysms in non-contrast CT scans, surgical planning, and predicting aortic aneurysm progression, and patient prognosis with high accuracy [293]. These potential advancements could be applied to aortopathies in RD, for which no definitive monitoring guidelines have been established, thereby enabling more accurate, personalized care and improved patient outcomes.

## 7. Conclusions

Aortopathies in rheumatic diseases encompass a wide range of aortic pathologies primarily driven by inflammatory processes, necessitating early recognition and aggressive management to mitigate severe cardiovascular complications. Due to the complexity and potential severity of aortic involvement, management should be personalized and often requires a multidisciplinary approach to improve outcomes. Regular follow-up and imaging are essential for monitoring disease progression and evaluating the efficacy of therapeutic interventions. Continued research and a personalized, patient-centered approach are essential to address the current evidence gap in clinical practice.

## Figures and Tables

**Table 1:** (**a**) Summary of inflammatory patterns of aortitis/periaortitis in rheumatic diseases References: entries are compiled from the primary sources cited in the Pathophysiology and Histopathology section [1,2,4,6,8,9,10]. (**b**) Histopathology of aortic abnormalities in rheumatic disease and prevalence of aortitis.

**(a)**			
**Predominant Inflammatory Composition**	**Histopathologic Pattern**	**Typical Histologic Features**	**Associated Rheumatic Diseases**
Epithelioid histiocytes and multinucleated giant cells	Granulomatous/giant cell pattern	Poorly formed granulomas, sometimes necrotizing	GCA; GPA, EGPA; clinically isolated aortitis
Epithelioid histiocytes and multinucleated giant cells	Granulomatous pattern	Compact, well-formed, non-necrotizing granulomas	Sarcoidosis
Epithelioid histiocytes and multinucleated giant cells	Variable granulomatous pattern	Well or poorly formed granulomas, occasionally necrotizing	RA; TA
Lymphocytes and plasma cells	Lymphoplasmacytic pattern	Dense lymphoplasmacytic infiltrate without a granulomatous component; in IgG4-related disease, abundant IgG4-positive plasma cells, predominantly adventitial involvement, dense sclerosis, and obliterative phlebitis	IgG4-RD, SLE, AS
Mixed inflammatory cells	Mixed inflammatory pattern	Polymorphous inflammatory infiltrate without granuloma formation	Cogan syndrome; BD; RP
**(b)**			
**Condition**	**Prevalence of aortitis**	**Findings**	
**GCA**	30–80%	Gross: wrinkled ‘tree bark’ appearance of intima Histology (Figure 2): granulomatous inflammation in media, often with necrosis; multinucleated giant cells; inflammatory infiltrate of vasa vasorum; laminar medial necrosis; irregular zonal areas of destroyed elastic laminae (best viewed with elastic stain)	
**Clinically Isolated Aortitis**	100%	Granulomatous, lymphoplasmacytic, or mixed inflammatory pattern	
**TA**	80%	Gross: wall thinning and intimal wrinkle in the acute phase; circumferential mural and intimal thickening in the chronic/late phase. Histology (Figure 2): like GCA in the acute phase; fibroplastic thickening of intima, dense fibrosis of media and adventitia in the chronic phase	
**GPA**	Rare	Geographic areas of necrotizing granulomatous inflammation in media with basophilic necrosis; neutrophilic microabscesses; small hyperchromatic “Wegener-type” multinucleated giant cells; may also involve vasa vasorum (Figure 2)	
**EGPA**	unknown	Necrotizing granulomatous aortitis similar to GPA; granulomatous inflammation mixed with eosinophils largely restricted to adventitia	
**RA**	1–5%	Lymphocytic or lymphoplasmacytic infiltrate in media and adventitia with medial degeneration Specific: rarely rheumatoid nodules (necrosis and palisading histiocytes)	
**SLE**	rare	Lymphocytic or lymphoplasmacytic infiltrate in the adventitia and media with medial degeneration	
**AS**	unknown	Focal inflammation of media with destruction of elastic and muscular tissue and fibrosis; intimal fibroplasia; fibrous thickening of adventitia Inflammatory narrowing of the vasa vasorum surrounded by lymphocytes and plasma cells	
**RP**	rare	Intimal fibrosis; mixed acute and chronic inflammatory infiltrate; destruction of medial elastic tissue and focal necrosis; microabscesses	
**BD**	<10%	Early: Mixed inflammatory pattern mainly in media and adventitia; vasa vasorum proliferation Late: Fibrous thickening of media and adventitia	
**Cogan’s syndrome**	10%	Neutrophilic microabscesses and mononuclear cell infiltrates of all vascular layers, predominantly in intima and media Occasional giant cells; loss of smooth muscle cells; degradation of elastic lamellae and collagenous matrix; Hypertrophy of fibrous connective tissue; focal necrosis with transmural wall destruction	
**Sarcoidosis**	rare	Non-necrotizing, well-formed, “sarcoid-like” granulomas at the media–adventitia junction with irregular areas of dense fibrosis.	
**SSc**	unknown	Increased collagen content, thickened adventitia, and smooth muscle layer; atherosclerosis (plasma cell infiltrate)	
**IgG4-related aortitis/peri-aortitis**	Up to 15%	Dense sclerosis associated with prominentIgG4-positive plasma cell infiltrates predominantly in adventitia (usually >50 IgG4+ plasma cells per high-power field; elevated IgG4:IgG ratio (>40%); storiform fibrosis; obliterative phlebitis; lymphoid follicles and perineural chronic inflammation	
**VEXAS Syndrome**	Rare	Unknown No characteristic histopathological pattern established. Temporal artery biopsy is usually unrevealing; a positive biopsy showing focal neutrophilic-predominant inflammation in the intima was reported in only 1 of 18 biopsied patients in a recent cohort	

GCA, giant cell arteritis; GPA, granulomatosis with polyangiitis; EGPA, eosinophilic granulomatosis with polyangiitis; RA, rheumatoid arthritis; TA, Takayasu arteritis; IgG4-RD, immunoglobulin G4-related disease; SLE, systemic lupus erythematosus; AS, ankylosing spondylitis; BD, Behçet disease; RP, relapsing polychondritis.

**Table 2:** Current classification of TA by types, its involvement, and the prevalence of each type Reference: classification and prevalence adapted from [11,12,13,14,15].

Type	Site of Aortic Involvement	Percentage of Patients
**I**	Branches of the aortic arch	10–50%
**II**	Ascending aorta, aortic arch, and its branches	1–19%
**IIb**	Ascending aorta, aortic arch, and its branches, and the thoracic descending aorta	0–10%
**III**	Thoracic descending aorta, abdominal aorta, and/or renal arteries	0–18%
**IV**	Abdominal aorta and/or renal arteries	0–29%
**V**	Combination of type IIb and IV	31–56%

**Table 3:** Summary of IgG4-aortitis and IgG4-CP features.

Feature	IgG4-Related Aortitis	IgG4-Related Chronic Periaortitis
**Prevalence of IgG4-related disease**	Approximately 8%	Approximately 20–36%
**Predominant vascular involvement**	Thoracic aorta > Abdominal aorta	Abdominal aorta > Thoracic aorta
**Patient characteristics**	Typically affects elderly patients; male or female predominance may vary	Typically affects elderly patients; more common in men
**Major complications**	Ascending aortic and aortic arch aneurysms; dissection is less common	Aortic dilatation or aneurysm in approximately 30% of cases
**Laboratory findings**	Elevated serum IgG4; elevated ESR and/or CRP may be present	Elevated serum IgG4; elevated ESR and/or CRP may be present

IgG4-RD, immunoglobulin G4-related disease; IgG4-CP, IgG4-related chronic periaortitis; ESR, erythrocyte sedimentation rate; CRP, C-reactive protein.

**Table 4:** Classification of aortic involvement in IgG4-RD based on imaging findings.

Type	Peng et al. [64]	Ozawa et al. [74]
**1**	Ascending aorta, aortic arch, or descending thoracic aorta	Localized infrarenal abdominal aorta
**2a**	Abdominal aorta	Extension into medium-sized arteries, such as the aortoiliac arteries
**2b**	Abdominal aorta and axillary artery	—
**2c**	Iliac artery	—
**3**	Thoracic and abdominal aorta	Extension into medium-sized arteries with additional separate involvement of the ascending aorta
**4**	—	Medium-sized vessels only
**5**	—	All other sites

**Table 5:** Key points for the differential diagnosis of aortitis/periaortitis among rheumatic diseases.

Disease	Typical Age/Sex & Clues	Aortic Segment & Pattern	Distinguishing Features
**Takayasu arteritis**	<40 y, female predominance	Whole aorta and branches; long concentric stenosis	Claudication, blood-pressure discrepancy; stenosis > aneurysm
**Giant cell arteritis**	>50 y; headache, jaw claudication, PMR	Thoracic aorta; wall thickening, later aneurysm	Temporal artery involvement; strong steroid response
**IgG4-related disease**	Older men; other organ involvement	Infrarenal abdominal aorta; periaortitis	Peri-aortic cuff, elevated IgG4, storiform fibrosis
**Behçet disease**	Young men; oral/genital ulcers, uveitis	Any segment; saccular aneurysm/pseudoaneurysm	Aneurysm > stenosis; pathergy; pulmonary artery aneurysms
**Ankylosing spondylitis**	Long-standing axial SpA	Aortic root/ascending aorta	Aortic root thickening and regurgitation
**Cogan’s syndrome**	Young adults; audiovestibular & ocular disease	Ascending aorta and arch	Interstitial keratitis with hearing loss
**VEXAS syndrome**	Men > 50 y; macrocytic anaemia, vacuoles	Large-vessel aortitis	Somatic UBA1 mutation; refractory inflammation
**Sarcoidosis**	Any age; pulmonary/lymph-node disease	Any segment; aneurysm or stenosis	Non-caseating granulomas in multiple organs

**Table 6:** Summary of indications, advantages, and limitations of imaging modalities used to evaluate aortic involvement in rheumatic diseases.

Modality	Primary Indication	Advantages	Limitations
**Ultrasound/Doppler (including echocardiography)**	First-line for suspected GCA (axillary/temporal arteries); detection of aortic root and valve disease, mural thickening, aneurysm, dissection	Portable, no ionizing radiation, real-time flow assessment with Doppler, widely available	Operator-dependent; limited for the thoracic aorta and deep vessels
**CT angiography (CTA)**	Rapid whole-aorta assessment of mural thickening (>2–3 mm), enhancement, stenosis/occlusion, aneurysm and dissection; follow-up	Widely available, fast, 3D reconstruction, quantifies disease extent and severity; perfusion CT can monitor activity	Ionizing radiation and iodinated contrast; limited temporal resolution; not portable
**MRI/MRA**	Preferred first-line for Takayasu arteritis; mural inflammation, edema and luminal change	No ionizing radiation; detects subtle wall edema; good soft-tissue contrast	Longer acquisition; unsuitable for unstable patients; contraindicated with certain implants
**18F-FDG PET (PET/CT, PET/MRI)**	Detection of active large-vessel inflammation; assessment of treatment response and relapse	High sensitivity (76%) and specificity (93%) for early inflammation; whole-body survey; co-registration improves yield	May not distinguish atherosclerosis from vasculitis; radiation; limited availability (PET/MRI); affected by hyperglycaemia/steroids
**Conventional (catheter) angiography**	Reserved for cases undiagnosed non-invasively or requiring revascularization	Gold standard for luminal detail; enables intervention	Invasive; detects luminal changes only, not the vessel wall; radiation and contrast

**Table 7:** Conventional and emerging laboratory biomarkers in the assessment of aortic involvement in rheumatic diseases.

Category	Biomarker	Role/Utility	Limitations
**Conventional acute-phase reactants**	ESR, CRP, leukocyte count	Widely available; used for initial assessment and monitoring of disease activity	Nonspecific; normal in ~40% of TA and ~22% of GCA during active disease; poor correlation with periaortitis
**Conventional acute-phase reactants**	Serum IgG4	Supports IgG4-related disease when >5× upper limit of normal	Variable; not sufficient for diagnosis; may be normal despite active disease
**Disease-specific serology/genetics**	ANCA; UBA1 genetic testing	Delineate ANCA-associated vasculitis; genetic testing for UBA1 mutations establishes VEXAS syndrome	Not aorta-specific; UBA1 testing indicated mainly in older men with hematologic features
**Emerging biomarkers**	IL-6	More sensitive than acute-phase reactants for active disease and flares in TA and GCA	Confounded by IL-6–targeted therapy (e.g., tocilizumab)
**Emerging biomarkers**	Pentraxin-3 (PTX3)	Independent of ESR/CRP; proposed marker of arterial inflammation	Conflicting data; larger cohorts failed to confirm correlation with activity
**Emerging biomarkers**	IL-17, IL-8, IL-18, MMP-2, MMP-9	Investigated in TA/GCA and vascular BD; MMP-2 linked to aneurysmal disease	Inconclusive or preliminary; not validated for routine use

## Data Availability

No new data were created or analyzed in this study. Data sharing is not applicable to this article.

## Abbreviations

The following abbreviations are used in this manuscript:

AAA	Abdominal aortic aneurysm
AAV	ANCA-associated vasculitis
ACR	American College of Rheumatology
AI	Artificial intelligence
ANA	Antinuclear antibody
ANCA	Antineutrophil cytoplasmic antibody
APS	Antiphospholipid syndrome
ARDs	Autoimmune rheumatic diseases
AS	Ankylosing spondylitis
AZA	Azathioprine
BD	Behçet’s disease
BiAATE	Bispecific autoantigen T-cell engager
CAPS	Catastrophic antiphospholipid syndrome
CAR	Chimeric antigen receptor
CHCC	Chapel Hill Consensus Conference
CI	Confidence interval
CP	Chronic periaortitis
CRP	C-reactive protein
CSA	Cyclosporine
CT	Computed tomography
CTA	Computed tomography angiography
CYC	Cyclophosphamide
DMARDs	Disease-modifying antirheumatic drugs
EGPA	Eosinophilic granulomatosis with polyangiitis
ESR	Erythrocyte sedimentation rate
EULAR	European Alliance of Associations for Rheumatology
EVAR	Endovascular aneurysm repair
FDA	Food and Drug Administration
FDG	Fluorodeoxyglucose
FDG-PET	Fluorodeoxyglucose positron emission tomography
FDG-PET/CT	Fluorodeoxyglucose positron emission tomography/computed tomography
GC	Glucocorticoids
GCA	Giant cell arteritis
GM-CSF	Granulocyte–macrophage colony-stimulating factor
GPA	Granulomatosis with polyangiitis
HLA	Human leukocyte antigen
IAA	Inflammatory abdominal aortic aneurysm
IgG4-RD	Immunoglobulin G4-related disease
IL	Interleukin
IST	Immunosuppressive therapy
IVIg	Intravenous immunoglobulin
LVV	Large-vessel vasculitis
MICA	Major histocompatibility complex class I chain-related gene A
MMF	Mycophenolate mofetil
MMP	Matrix metalloproteinase
MPA	Microscopic polyangiitis
MR	Magnetic resonance
MRA	Magnetic resonance angiography
MRI	Magnetic resonance imaging
MTX	Methotrexate
NSAIDs	Nonsteroidal anti-inflammatory drugs
OR	Odds ratio
PA	Periaortitis
PCI	Percutaneous coronary intervention
PET	Positron emission tomography
PET-CT	Positron emission tomography–computed tomography
PET-MRI	Positron emission tomography–magnetic resonance imaging
PMR	Polymyalgia rheumatica
PTX3	Pentraxin-3
RA	Rheumatoid arthritis
RD	Rheumatic disease
RP	Relapsing polychondritis
RPF	Retroperitoneal fibrosis
sIL-2R	Soluble interleukin-2 receptor
SLE	Systemic lupus erythematosus
SpA	Spondyloarthropathy
SS	Sjögren’s syndrome
SSc	Systemic sclerosis
SSZ	Sulfasalazine
TA	Takayasu arteritis
TAA	Thoracic aortic aneurysm
TEVAR	Thoracic endovascular aortic repair
TNF-α	Tumor necrosis factor-alpha
TNFi	Tumor necrosis factor inhibitor
TTE	Transthoracic echocardiogram
UBA1	Ubiquitin-like modifier activating enzyme 1
VEXAS	Vacuoles, E1 enzyme, X-linked, autoinflammatory, somatic syndrome
